# Interaction between the flagellar pocket collar and the hook complex *via* a novel microtubule-binding protein in *Trypanosoma brucei*

**DOI:** 10.1371/journal.ppat.1006710

**Published:** 2017-11-01

**Authors:** Anna Albisetti, Célia Florimond, Nicolas Landrein, Keni Vidilaseris, Marie Eggenspieler, Johannes Lesigang, Gang Dong, Derrick Roy Robinson, Mélanie Bonhivers

**Affiliations:** 1 University of Bordeaux, Microbiologie Fondamentale et Pathogénicité, UMR 5234, Bordeaux, France; 2 CNRS, Microbiologie Fondamentale et Pathogénicité, UMR 5234, Bordeaux, France; 3 Max F. Perutz Laboratories, Medical University of Vienna, Vienna, Austria; University of Edinburgh, UNITED KINGDOM

## Abstract

*Trypanosoma brucei* belongs to a group of unicellular, flagellated parasites that are responsible for human African trypanosomiasis. An essential aspect of parasite pathogenicity is cytoskeleton remodelling, which occurs during the life cycle of the parasite and is accompanied by major changes in morphology and organelle positioning. The flagellum originates from the basal bodies and exits the cell body through the flagellar pocket (FP) but remains attached to the cell body *via* the flagellum attachment zone (FAZ). The FP is an invagination of the pellicular membrane and is the sole site for endo- and exocytosis. The FAZ is a large complex of cytoskeletal proteins, plus an intracellular set of four specialised microtubules (MtQ) that elongate from the basal bodies to the anterior end of the cell. At the distal end of the FP, an essential, intracellular, cytoskeletal structure called the flagellar pocket collar (FPC) circumvents the flagellum. Overlapping the FPC is the hook complex (HC) (a sub-structure of the previously named bilobe) that is also essential and is thought to be involved in protein FP entry. BILBO1 is the only functionally characterised FPC protein and is necessary for FPC and FP biogenesis. Here, we used a combination of *in vitro* and *in vivo* approaches to identify and characterize a new BILBO1 partner protein—FPC4. We demonstrate that FPC4 localises to the FPC, the HC, and possibly to a proximal portion of the MtQ. We found that the C-terminal domain of FPC4 interacts with the BILBO1 N-terminal domain, and we identified the key amino acids required for this interaction. Interestingly, the FPC4 N-terminal domain was found to bind microtubules. Over-expression studies highlight the role of FPC4 in its association with the FPC, HC and FPC segregation. Our data suggest a tripartite association between the FPC, the HC and the MtQ.

## Author summary

*Trypanosoma brucei* is the parasite responsible for human African trypanosomiasis, a disease also known as sleeping sickness. African trypanosomiasis is present in Sub-Saharan Africa and transmitted by infected tsetse flies. This devastating disease is lethal if untreated, making it important to understand and characterise the basic biology of the pathogen. During the *T*. *brucei* cell cycle, organelle positioning and segregation show a high degree of coordination and control. As such, *T*. *brucei* is a highly polarised cell with the transition zone of the flagellum present inside an invagination of the pellicular membrane called the flagellar pocket (FP). The FP is the main site of traffic into and out of the cell. At the exit point of the flagellum is a cytoskeletal structure called the flagellar pocket collar (FPC). One component of the FPC, BILBO1, has been characterised as essential for the FP biogenesis and cell survival. Here, we identify a new partner of BILBO1 called FPC4, and determine the domains involved in the BILBO1-FPC4 interaction. We further highlight the role of FPC4 in the segregation of the FPC and in the interplay between the FPC and other essential cytoskeletal structures.

## Introduction

Cell polarisation requires precise positioning and connection of organelles during the cell cycle [1,2]. This applies to the unicellular, pathogenic parasite *Trypanosoma brucei*, the aetiological agent of human African trypanosomiasis [3]. In *T*. *brucei*, organelle positioning and segregation during the cell cycle show a high degree of coordination and control [4–7]. Essential for pathogenicity, cytoskeleton remodelling occurs also during the parasite life cycle with major morphological alterations and changes in organelle positioning [8–11].

The *T*. *brucei* characteristic fusiform shape is mainly maintained by a sub-pellicular microtubule-based cytoskeleton [12]. The flagellum extends from the basal body tethered to the kinetoplast (the mitochondrial genome) [13,14], and exits the cell through the flagellar pocket (FP), an invagination of the plasma membrane. It then runs along the length of the cell whilst remaining attached to the cell body *via* the flagellum attachment zone (FAZ), a complex structure that mediates lateral attachment of the flagellum [12,15,16]. A set of four specialised microtubules (the microtubule quartet MtQ) nucleates at the basal bodies and extends around the FP, inserts into the sub-pellicular microtubules array and runs as part of the cytoplasmic portion of the FAZ up to the anterior end of the cell body [17–19]. MtQ polarity is considered to be opposite to the polarity of the sub-pellicular microtubules (the latter having their + end towards the posterior end of the cell), but the same polarity as the axoneme microtubules that have their + end towards the distal tip of the flagellum [4]. The functional role of the MtQ is still unknown.

The membrane of the FP is devoid of sub-pellicular microtubules (MTs) and is the sole site for endo- and exocytosis processes. As such, the FP is a key player in protein trafficking, cell signalling and immune evasion through the removal of surface-bound host immune factors [20–23]. The FP encircles the flagellum at the FP neck, the latter being maintained by the flagellar pocket collar (FPC), a cytoskeletal structure situated at the exit point of the flagellum [24,25,19]. The Golgi apparatus is precisely positioned between the kinetoplast and the nucleus in procyclic form (PCF—found in the tsetse fly) and is considered to be physically connected to the FP or the neck region of the FP [19,26,27]. Overlapping the FPC is the bilobe, an essential structure thought to be involved in protein entry in the FP [28,29].

The bilobe is a cytoskeleton-associated structure that overlaps the FPC and contains the cell cycle progression regulators centrin2, centrin4, the Polo-like kinase *Tb*PLK that is associated transiently [28,30–32], and the tubulin co-factor TBCCD1 [33], together with other uncharacterised proteins [34–38]. RNAi knockdown (RNAi) of any of the above-named proteins leads to defects in organelle duplication, segregation and cytokinesis, and disorganisation of the bilobe structure [28,30,33]. The bilobe is composed of two domains; the hook complex (HC), and the centrin arm, which is adjacent to the stem of the HC. The MtQ threads between these two structures [29]. To date, only three HC-specific proteins have been characterised as essential for cell survival in PCF or bloodstream form (BSF, found in the mammalian hosts). The tubulin co-factor TBCCD1 is associated with filament-based structures in the cytoskeleton and is essential in PCF [33]. The Leucine-Rich Repeat Protein LRRP1 is a Ran regulator with essential role in FAZ assembly, flagellum inheritance, and cell division and is essential in PCF [39,40]. The Membrane Occupation and Recognition Nexus protein 1 (MORN1) may be involved in the endomembrane balance and in facilitating protein entry into the FP and has been shown to be essential in BSF [41,42]. LRRP1 and MORN1 co-localise at the HC and are the landmarks of the HC. However, at the “hook” region of the HC, both proteins are super-imposed on top of the FPC [43].

The FPC plays essential roles in the biogenesis and function of the FP and thus in the viability of PCF parasites [25]. However, the mechanisms behind FPC biogenesis and function remain elusive, mostly due to poor knowledge of its molecular composition. The FPC is also a complex structure, and in addition to its ring/horse-shoe shape and attachment to the flagellum, it is attached to the sub-pellicular microtubule cytoskeleton [19,24,25]. To date, BILBO1 is the only FPC component that has been functionally characterised, and importantly, it is required for FPC biogenesis in PCF [44]. RNAi of BILBO1 abolishes the biogenesis of a new FPC, FP and FAZ, and the newly formed flagellum is positioned at the extended posterior end of the cell and is detached from the cell body. This means that the FPC is clearly required for the biogenesis of numerous structures and functions in the cell.

BILBO1 is a structural protein with four main functional domains. The N-terminal domain (NTD) folds into a ubiquitin-like fold and deletion or mutation of key residues within this domain affects cell viability, suggesting that it could be involved in the interaction with partner proteins. Two EF-hand domains follow the NTD, then a central coiled-coil (CC) and a C-terminal leucine zipper (LZ). The LZ is necessary, but not sufficient, for FPC targeting. The CC allows the formation of antiparallel dimers that can extend into filaments by interdimer interaction between adjacent LZ. Deletion of the NTD or mutation of key residues in either the NTD or the EF-hand domains influence the shape of the polymers formed and affect cell viability, suggesting that BILBO1 forms a molecular frame on which other FPC proteins can interact with [25,45,46,44].

The FPC and the HC remain super-imposed during the trypanosome cell cycle and are in close proximity with the microtubule cytoskeleton and the MtQ [43]. Indeed tomographical data demonstrate that the MtQ actually traverses the FPC [19], and the shank of the HC is adjacent to, if not part of, the MtQ [43]. Therefore, it is thus reasonable to imagine that one or more proteins can link the FPC and the HC to the MtQ.

To understand more about FPC composition and function, we carried out a genomic yeast two-hybrid screen using BILBO1 as bait. Among several putative BILBO1 binding partners, we identified Tb927.8.6370, which was given the annotation FPC4 for Flagellar Pocket Collar protein 4. Using a series of biochemical and cellular biology approaches, we demonstrate that FPC4 is a *bona fide* BILBO1 partner and is a microtubule-binding protein that potentially plays a role in FPC segregation.

## Results

### Identification of FPC4, a BILBO1 protein partner

Yeast two-hybrid (Y2H) is a well-established technique for analysing and determining protein-protein interactions. Using BILBO1 as bait in a *T*. *brucei* 927 genomic Y2H screen (Hybrigenics), we identified several putative partners. Among them was Tb927.8.6370, which we named FPC4—for Flagellar Pocket Collar protein 4. FPC4 is a 48.9 kDa protein consisting of 444 amino acids with a calculated pI of 10.61. It is encoded by a kinetoplastid specific gene whose synteny is conserved in trypanosomes, but not in *Leishmania* species.

The BILBO1-binding domain of FPC4 that was identified in the Y2H screen lies within the region of amino acids (aa) 357 to 444 (from now on named BILBO1 binding domain—B1BD). FPC4 primary sequence analysis did not predict any functional domains beside a putative poly-proline motif between aa 28–40 potentially for SH3 binding, and a coiled-coil domain between aa 218–252 (embnet.vital-it.ch/software/COILS_form.html [47]).

### Identification of the domains involved in BILBO1-FPC4 interaction

**Fig 1:**
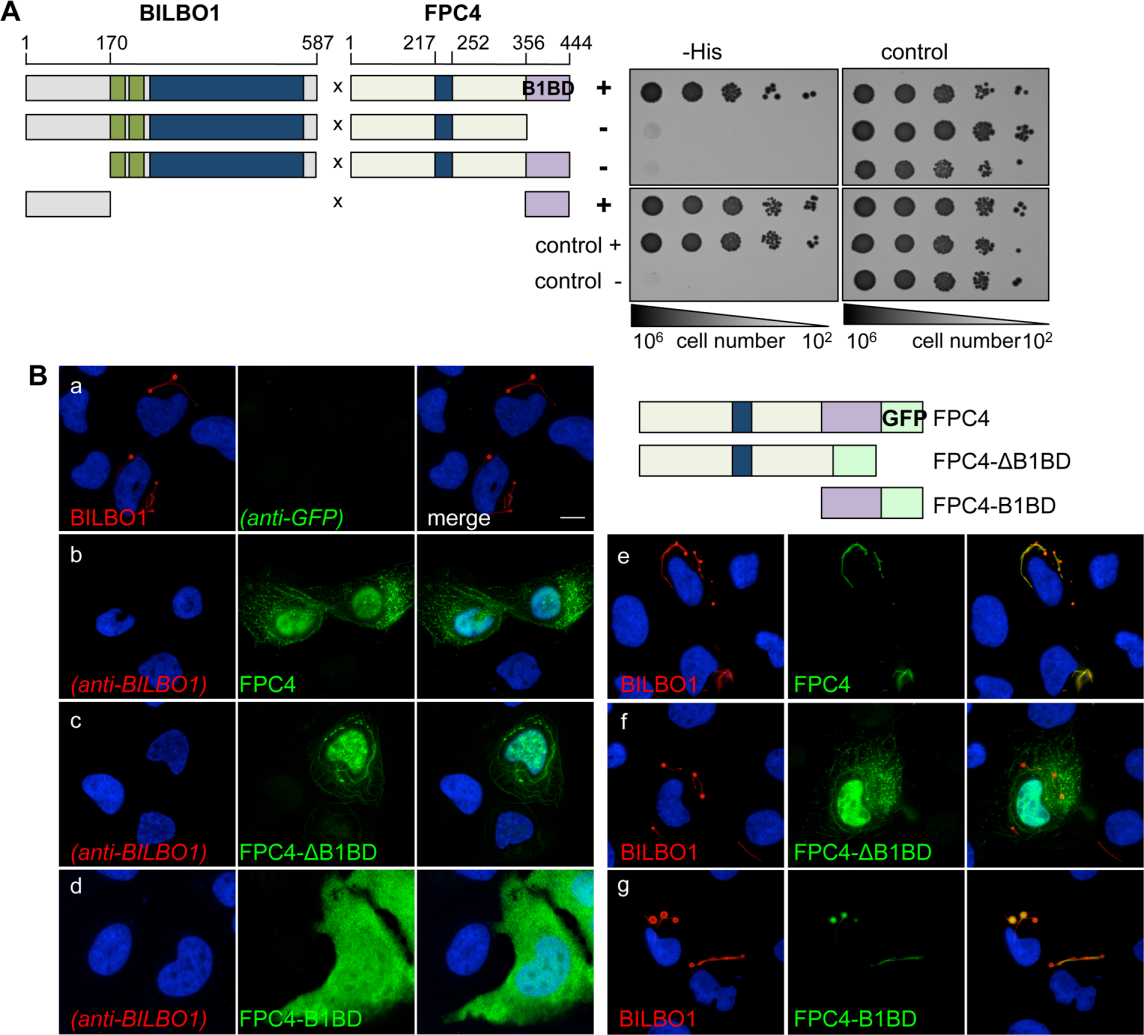
BILBO1—FPC4 interaction involves their N-terminal and C-terminal domains respectively. (A) BILBO1-FPC4 yeast two-hybrid interaction test. Left panel: Schematic overview of BILBO1 and FPC4 domains and of the combinations tested by Y2H. The EF-hand calcium binding sites of BILBO1 are represented in green and the coiled-coil domains in BILBO1 and FPC4 are represented in blue. The FPC4 B1BD is represented in violet. Right panel: The interactions tested were probed on–histidine selective medium (-His) and on growth control medium (control) (n = 3). Positive control involved p53 and T-antigen, whereas negative control involved Lamin and T-antigen. (B) Heterologous expression and co-expression in U-2 OS cells of FPC4 and FPC4 truncations fused to a C-terminal GFP tag, and of BILBO1. BILBO1 (a), FPC4 (b), FPC4-ΔB1BD (c) and FPC4-B1BD (d) were expressed alone and cells were probed with anti-BILBO1 (red) and anti-GFP (green). In e, f, g, cells were expressing BILBO1 + FPC4, BILBO1 + FPC4-ΔB1BD, and BILBO1 + FPC4-B1BD respectively and were also probed with anti-BILBO1 (red) and anti-GFP (green). Cells were extracted before labelling, except in (d) to show the cytoplasmic localisation of FPC4-B1BD, which is not visible on extracted cells. The transfections were performed more than three independent times. Nuclei were DAPI stained (blue). Scale bar represents 10 μm.

The interaction between FPC4 and BILBO1 *via* the FPC4 B1BD was confirmed by yeast two-hybrid interaction tests using full-length BILBO1 and full-length FPC4 as well as truncations of both proteins (Fig 1A). Notably, BILBO1-FPC4 interaction was abolished when either the N-terminal domain of BILBO1 (NTD, aa 1–170) or the FPC4 B1BD domain (FPC4-ΔB1BD, aa 1–356) were deleted. Further, the BILBO1 NTD alone interacts with FPC4 B1BD, thus demonstrating that the N-terminal domain of BILBO1 and the FPC4 B1BD are required and sufficient for BILBO1-FPC4 interaction.

It was previously described that BILBO1 forms polymers *in vitro* [46] or *in vivo* in mammalian cells [44]. We simultaneously expressed BILBO1 and FPC4-GFP (or several GFP tagged truncations of FPC4) in U-2 OS cells to further characterise the BILBO1-FPC4 interaction (Fig 1B). Expression of BILBO1 alone forms polymers with globular structures at the extremities, as previously described [44] (Fig 1Ba). FPC4-GFP or FPC4 minus the B1BD domain (FPC4-ΔB1BD-GFP) was observed in linear filaments (Fig 1Bb, c), whilst FPC4-B1BD-GFP was cytoplasmic (Fig 1Bd). Some nuclear labelling was also observed, most probably due to a weak bipartite nuclear localisation sequence between the amino acids (aa) 120–154 that was predicted by NLS mapper [48]. Since FPC4 never localises to the nucleus in trypanosomes, we did not further address the nuclear localisation in the U-2 OS cells (see below). When co-expressed with BILBO1, FPC4-GFP or GFP-tagged B1BD (FPC4-B1BD-GFP) co-localised with BILBO1 polymers, whilst FPC4 deleted of its B1BD (FPC4-ΔB1BD-GFP) was not recruited to the BILBO1 assembly (Fig 1B e—g). Consequently, using two different *in vivo* heterologous systems (yeast and U-2 OS cells), we were able to demonstrate that FPC4 is a BILBO1 partner protein and that the aa 357–444 domain of FPC4 is involved in its interaction with the BILBO1 NTD.

**Fig 2:**
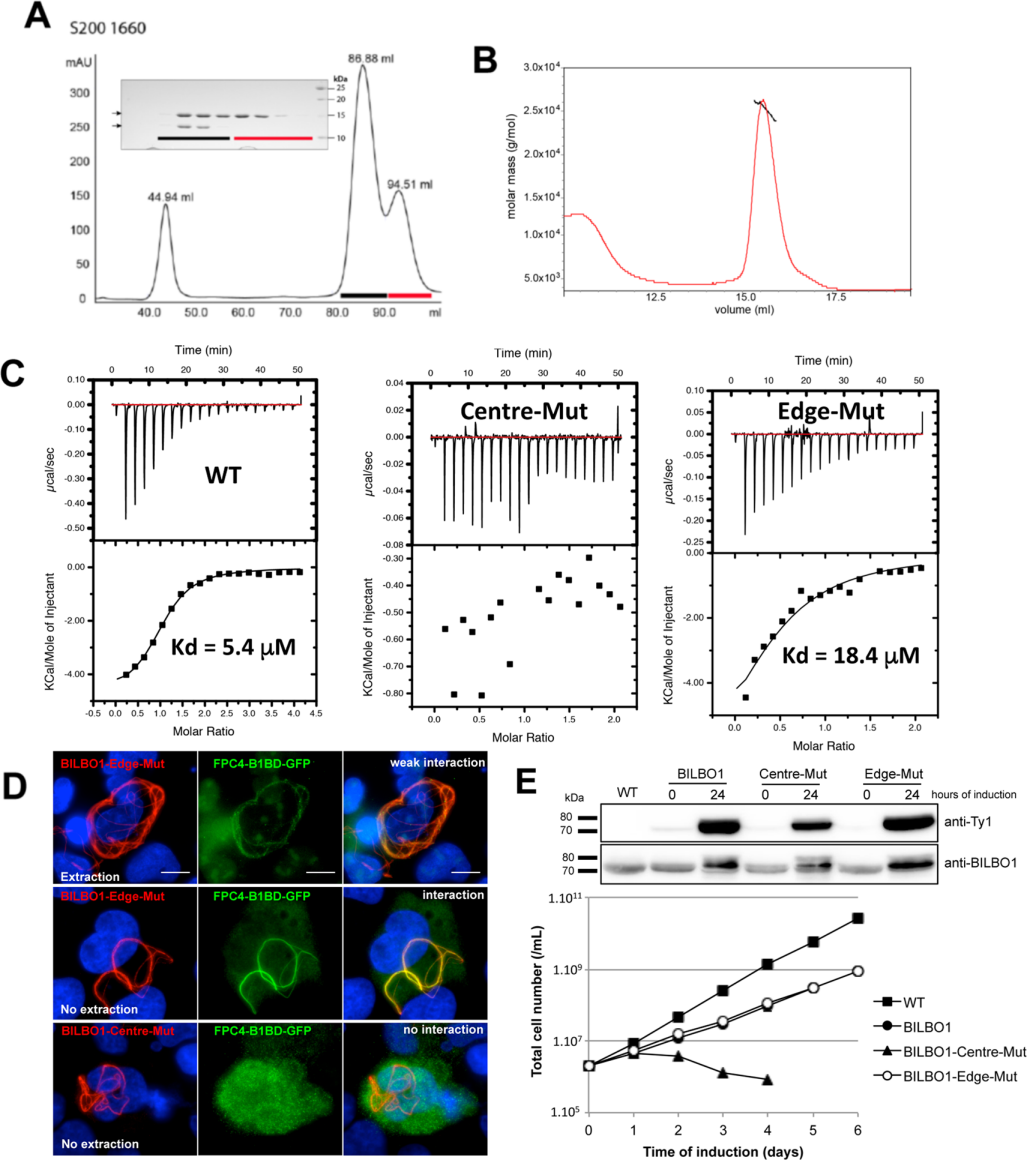
The formation of a stable complex between the BILBO1-NTD and FPC4-B1BD depends on the residues Y87 and F89 on the BILBO1-NTD. (A) SEC elution profile (S-200 16/60) of the mixture of purified BILBO1-NTD’ (aa1-120) and FPC4-B1BD (aa357-440). Both proteins were co-eluted in the elution peak of 86.88 ml. The 44.95 peak is the void volume. Inset: Coomassie-stained SDS-PAGE gel showing proteins in the fractions indicated with the coloured bars in the elution profile. Upper arrow indicates the BILBO1-NTD’ and lower arrow indicates FPC4-B1BD. (B) SLS results of the complex of BILBO1-NTD’ and FPC4-B1BD. The apparent molecular mass of 24.9 kDa indicates the formation of a hetero-dimer. (C) ITC analysis showing that interaction with FPC4-B1BD is partially reduced in BILBO1-Edge-Mut but completely abolished in BILBO1-Centre-Mut. (D) Co-expression in U-2 OS cells and immunolabelling on permeabilised cells (no extraction) or extracted cells (extraction) of FPC4-B1BD-GFP (anti-GFP, green) with either BILBO1-Centre-Mut or BILBO1-Edge-Mut (anti-BILBO1, red). Nuclei were DAPI stained. Scale bar represents 10 μm. The experiments were performed more than three independent times. (E) WB and growth curves for the PCF WT and induced cell lines for the expression of Ty1-tagged BILBO1, BILBO1-Centre-Mut, and BILBO1-Edge-Mut. Error bars represent the standard error from 3 independent experiments (and are smaller than the data point mark).

To assess the stoichiometry of the FPC4-B1BD–BILBO1-NTD complex, we co-expressed maltose binding protein (MBP)-tagged FPC4-B1BD (aa 357–440) and _6His_BILBO1-NTD’ (aa 1–120) in *E*. *coli*, and the complex was purified by nickel affinity chromatography. After removing the MBP and the 6xHis tags, the purified complex was checked by size exclusion chromatography (Superdex-200 16/60), which resulted in two elution peaks (Fig 2A). SDS-PAGE analysis (inset) indicated that the peak at the retention volume of 86.88 ml corresponded to the BILBO1-NTD’–FPC4-B1BD complex, and that the peak at the retention volume of 94.51 ml corresponded to the BILBO1-NTD’ only. Analysis by static light scattering (SLS) showed that the complex had a molecular mass of 24.9 ± 1.0 kDa, which corresponded to one molecule of BILB01-NTD' (aa 1–120, MW = 14.1 kDa) and one molecule of FPC4-B1BD (aa 357–440) (MW = 10.0 kDa) (Fig 2B).

It was reported previously that a series of aromatic and hydrophobic residues forms a crater-like structure on the solvent-exposed face of the conserved surface patch of BILBO1-NTD. Previously described mutations of the residues that form the “rim” (F12A/K15A/K60A/K62A, previously reported as mut1) or the “bottom” of the crater-like structure (W71A/Y87A/F89A, previously reported as mut2) impaired BILBO1 function, demonstrating that they are essential for FPC function [45]. To further characterise the interaction between BILBO1-NTD and FPC4-B1BD, we generated two novel sets of mutations where the “rim” residues Lys-60 and Lys-62 (being half of the rim mutation mut1 and named Edge-Mut) and the “bottom” residues Trp-87 and Phe-89 (named Centre-Mut) were substituted with alanine. After expression in bacteria and purification, we assessed by isothermal titration calorimetry (ITC) the interaction of the mutant proteins with the FPC4-B1BD (Fig 2C). Wild-type (WT) BILBO1-NTD bound strongly to the FPC4-B1BD (dissociation constant Kd ≈ 5μM). Edge-Mut partially reduced the binding affinity (Kd ≈ 18μM), whilst Centre-Mut completely abolished the interaction. Additionally, FPC4-B1BD and BILBO1 Centre-Mut or Edge-Mut were co-expressed and immuno-labelled in U-2 OS cells (Fig 2D). Interestingly, the polymers formed by BILBO1-Edge-Mut and BILBO1-Centre-Mut were slightly different to non-mutated BILBO1 alone, because no annular termini were observed as described in Fig 1B and in [44]. This suggests that mutation of the residues on the BILBO1-NTD involved in FPC4 binding modifies the polymer shape formed by BILBO1. Similar polymer shape modification was previously shown in trypanosome when mut1 and mut2 mutants were expressed [45]. When co-expressed with BILBO1-Centre-Mut, FPC4-B1BD was not associated with the BILBO1 polymers, but rather gave a cytosolic pattern (Fig 2D). Results of co-expression of FPC4-B1BD and BILBO1-Edge-Mut are consistent with the ITC data (Fig 2D). Using western-blotting we next assessed the expression of the Ty1-tagged Centre-Mut and Edge-Mut BILBO1 in trypanosomes upon induction with tetracycline (Fig 2E, upper panel), and followed cell growth (Fig 2E, lower panel). Induction of the expression of BILBO1 slightly reduced cell growth as previously described [44,45]. However, expression of Centre-Mut was lethal after 2 days of induction, whereas Edge-Mut did not affect cell growth.

These *in vitro* (Chromatography, SLS, ITC) and *in vivo* data (expression of mutated BILBO1 in U-2 OS cells and in trypanosomes) demonstrate that residues Trp-87 and Phe-89 are critical for the interaction between BILBO1-NTD and FPC4-B1BD. Taken together, these data demonstrate that the N-terminal domain of BILBO1 and the FPC4-B1BD can form a complex with a 1:1 stoichiometry, which is required and sufficient for the BILBO1-FPC4 interaction.

### FPC4 is a hook complex and an FPC protein

To localise FPC4 in the trypanosome, we produced a rat antibody raised against recombinant full-length FPC4 (anti-FPC4). We tested the specificity of anti-FPC4 by probing *T*. *brucei* whole cell extracts and extracts of bacteria expressing recombinant _6His_FPC4 or purified histidine-tagged FPC4 aa 1–260 and FPC4 aa 357–440 by western-blot (WB) (S1A, S1B and S1C Fig). In these blots, the anti-FPC4 polyclonal antibody recognizes the full-length and the truncated FPC4 (aa 1–260) purified proteins but not the FPC4 aa 357–440. The specificity of the anti-FPC4 antibody was also tested by immunofluorescence on FPC4 RNAi induced cells (S2 Fig). Unfortunately, neither the anti-FPC4 nor the anti-myc antibodies were able to detect the endogenous FPC4 (S1B Fig) or endogenously myc-tagged FPC4 (S1D Fig) respectively from trypanosome cell extracts. However, the proteins could be detected when over-expressed in trypanosomes.

**Fig 3:**
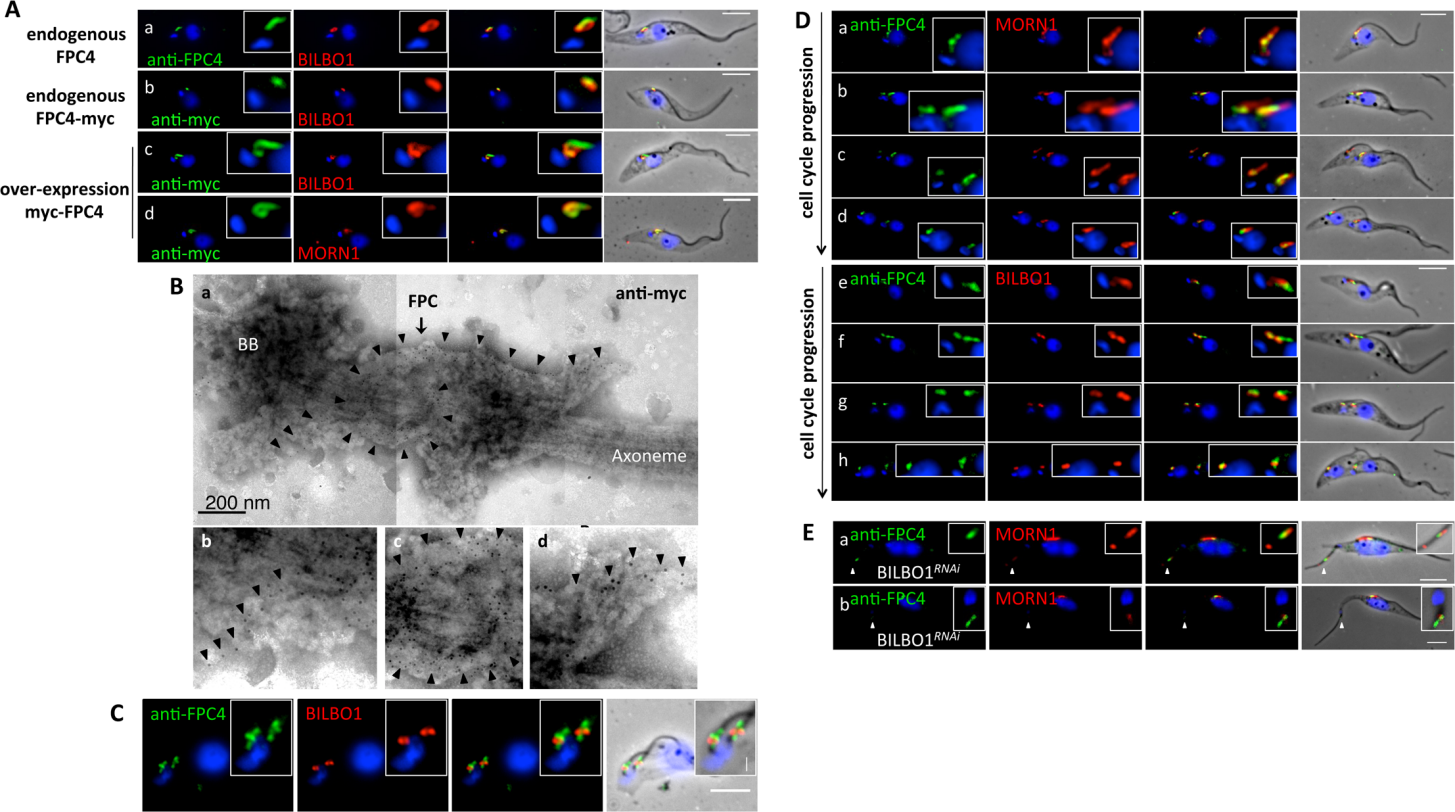
FPC4 is a hook complex and an FPC protein. (A) (a) Immunolabelling of endogenous FPC4 on cytoskeleton extracted cells from *Tb*427 29–13 cells using the rat anti-FPC4 antibody. (b) Cytoskeletons from the cell line expressing endogenously myc-tagged FPC4 probed with anti-myc. (c and d) Cytoskeletons from the cell line overexpressing myc-FPC4 using anti-myc. Co-labelling with anti-BILBO1 (a, b, c) or anti-MORN1 (d). (B) Anti-myc immuno-electron microscopy on flagella isolated from myc-FPC4 expressing cells (a), and the respective enlargements (b, c and d). (C) FPC4 immunolabelling using anti-FPC4 on CK extracted *Tb*427 90–13 BSF cells. (D) Co-labelling on cytoskeleton extracted *Tb*427 29–13 cells using anti-FPC4 and anti-MORN1 (a-d), and rat anti-FPC4 and anti-BILBO1 (e-h). (E) BILBO1^*RNAi*^ was induced for 36 h and cells were detergent extracted and probed with rat anti-FPC4 (green) and anti-MORN1 (red). The white arrowheads indicate the area that is enlarged in the zoom image and highlight the labelling of FPC4 and MORN1 within the new detached flagellum (a) and at the base of the axoneme of the new detached flagellum (b). Kinetoplasts and nuclei were DAPI stained in A, C, D and E. Scale bars in A, C, D, and E represent 5 μm. In A c and d where the cells were induced for 24 h. All the experiments were performed more than three independent times.

Considering that FPC4 and BILBO1 interact *in vitro* and in the U-2 OS cells, our co-labelling immunofluorescence experiments using anti-BILBO1 and anti-FPC4 on *T*. *brucei* cytoskeletons (CK) revealed, surprisingly, merely a close localisation of the two proteins (Fig 3Aa). This was also observed using anti-myc in the cell line expressing endogenous C-terminal myc-tagged FPC4 (Fig 3Ab). Clear co-localisation of FPC4 and BILBO1 was however observed in a tetracycline-inducible cell line that was over-expressing N-terminal myc-tagged FPC4 (Fig 3Ac). The structure highlighted by the anti-FPC4 and the anti-myc labelling resembled the shank and the hook of the HC; indeed, FPC4 and MORN1 co-localised as seen in Fig 3Ad and 3D a-d, suggesting that FPC4 is a component of the HC.

Unfortunately, endogenous FPC4 or myc-FPC4 were not detectable by immuno-gold electron microscopy (iEM). However, iEM on flagella, derived from PCF cells over-expressing myc-FPC4, showed that FPC4 localised on the HC (Fig 3B). Immunofluorescence on CK from BSF using the anti-FPC4 showed a clear labelling proximal and distal to the FPC (Fig 3C) suggesting a better accessibility for the antibody or higher expression of FPC4 in BSF. Unfortunately, as WB does not detect the endogenous protein, we could not compare protein expression levels between BSF and PCF that perhaps could explain the more intense IF labelling in BSF. In PCF, endogenous FPC4 was detectable by immunofluorescence at every stage of the cell cycle and was present on the old and the new FPC and HC, and always associated with MORN1 and BILBO1 (Fig 3D a-d) and (Fig 3D e-h).

During the later stages of this FPC4 project, a novel 10xTY1 endogenous tagging plasmid was developed by the Gull laboratory (University of Oxford, England, U.K). This plasmid was generously donated to our laboratory. We used the 10xTY1 plasmid to endogenously tag FPC4 in PCF and from the subsequent clonal cell lines we obtained excellent anti-TY1 immuno-fluorescence signal. Further, we could detect 10xTY1-FPC4 by western blotting (S1E Fig) demonstrating a better detection using the 10xTY1 tag over the myc-tag or over the anti-FPC4 polyclonal antibody. This increased detection permitted us to also obtain immuno-gold labelling (see below). The clearer and better 10xTY1 signal, compared to other tags, confirms the FPC4 localisation overexpression data (see below).

**Fig 4:**
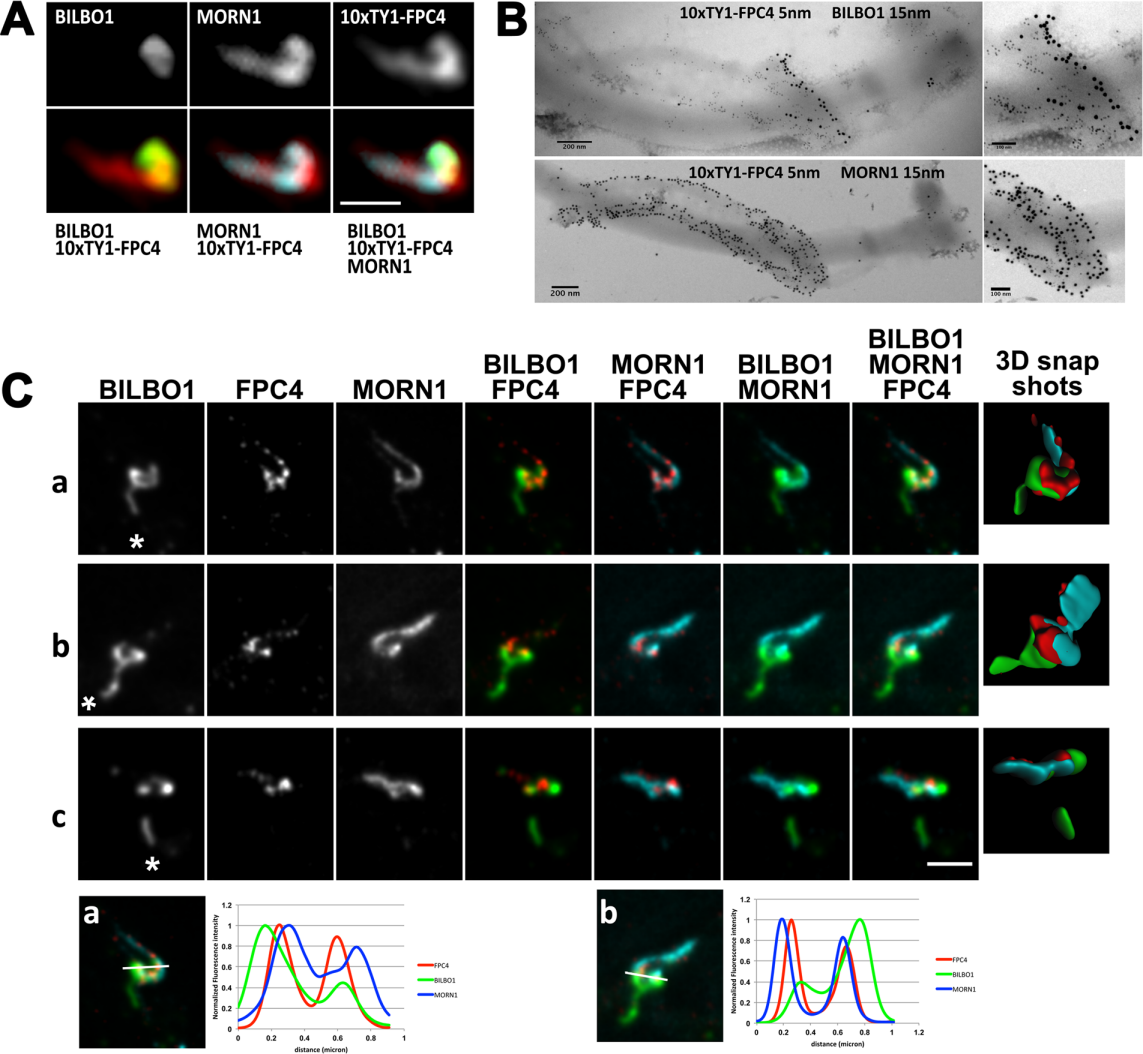
Immuno-localisation of endogenously tagged 10xTY1-FPC4, BILBO1 and MORN1. (A) Wide-field microscopy observation of the triple labelling of BILBO1, MORN1 and FPC4 on cytoskeleton from a SmOxP427 cell line expressing endogenously 10xTY1 tagged FPC4. (B) Immuno-electron microscopy on flagella labelled with anti-TY1 for FPC4 (5 nm gold beads), anti-BILBO1 (15 nm gold beads), and anti-MORN1 (15 nm gold beads). (C) STED confocal microscopy views of the co-labelling of BILBO1, MORN1 and FPC4 on cytoskeletons from a SmOxP427 cell line expressing endogenously 10xTY1 tagged FPC4 (a-c). The asterisks indicate the kinetoplast position relative to the structure. The snapshots of the 3D reconstructions show the close proximity of BILBO1 (green), FPC4 (red) and MORN1 (cyan). (D) Normalised intensities Plot corresponding to the section indicated by the white lines (a, b) indicate the co-localisation of the proteins. In A and B, scale bars represent 1 μm.

Our endogenously N-terminal 10xTY1 tagged FPC4 (10xTY1-FPC4) permitted labelling of FPC4, whilst also probing cells with anti-BILBO1 and anti-MORN1. This was done to further analyse FPC4 localisation using wide-field and STimulated Emission Depletion (STED) confocal microscopy. Wide-field immunofluorescence analysis confirmed that 10xTY1-FPC4 co-localises on the shank and hook of the HC with MORN1, but it also co-localises with BILBO1 on the FPC (Fig 4A). Immuno-electron microscopy data indicated that 10xTY1-FPC4 localised above BILBO1, and co-localises with MORN1 (Fig 4B). Results of STED analysis confirmed this data and further described FPC4 as a series of substructures regularly positioned close to the MORN1-labelled shank and hook of the HC. Moreover, MORN1 and FPC4 labelling showed close localisation on the hook region of the HC where it overlaps with, and is on top of, BILBO1 (Fig 4C). Three-dimensional reconstructions of all three immunolabelling signals show the close proximity of the three proteins (Figs 4C and 3D snapshots). Normalised intensity profile plots also highlight the proximity of these proteins, and show that FPC4 is sandwiched between MORN1 and BILBO1 (Fig 4D). The partial superposition of the peaks confirmed the interplay between the HC and the FPC. Interestingly, under the conditions used for STED, BILBO1 also appeared to be on the MtQ between the FPC and the basal bodies (Fig 4C).

Taken together, these data indicate that FPC4 is a cytoskeletal protein expressed in PCF and BSF parasites. FPC4 localises mainly at the HC together with MORN1, but is also present close to BILBO1 at the FPC, which suggests that the HC and the FPC share and overlap with FPC4. High-resolution STED analysis also showed that the FPC4-labelled region of the HC overlaps the FPC, and FPC4 is sandwiched at discrete points between BILBO1 and MORN1 suggesting that FPC4 is in simultaneous contact with MORN1 and BILBO1 and may be able to connect or link the FPC to the HC.

### The localisation of FPC4 and MORN1 depends on the presence of BILBO1

In PCF, BILBO1 RNAi knockdown induces new flagellum detachment from the cell body but it remains anchored to the cell through its basal body. BILBO1 knockdown also induces new flagellum relocation to the posterior end of the cell as well as absence of new FP and FPC. Moreover, the FAZ structure is affected because no new FAZ is associated with the new flagellum [25]. Immunofluorescence assays show that FPC4 localisation is also affected in BILBO1^*RNAi*^ cells (Fig 3E). In cells displaying the typical BILBO1^*RNAi*^ phenotype, anti-FPC4 antibody labelled the old FPC (where the old FPC remains present or intact). In these cells, FPC4 was detected in 70% of the new-detached flagella (n = 205). The flagella, however, did not have an FPC because induction of BILBO1 RNAi prevents new FPC formation [25]. In 82% (n = 72) of these new flagella, FPC4 labelling localised to the base of the axoneme. Importantly, in 18% of the new flagella that were labelled, the FPC4 labelling could be observed at foci along the length of the flagellum, suggesting that FPC4 was able to traffic into the flagellum in absence of the FPC. A similar labelling was observed for MORN1 in 51% of the new-detached flagella (n = 45). In these flagella, 78% had a signal at the base of the axoneme, and 22% were labelled within foci on the flagellum (n = 23). These data demonstrate that the correct localisation of FPC4 and MORN1 depends directly or indirectly on the presence of BILBO1. One possible hypothesis to explain this could be that in the absence of the FPC or MtQ in BILBO1^*RNAi*^ cells, FPC4 enters the flagellum and binds to axoneme MT. Furthermore, FPC4 and MORN1 both co-localise in the new detached flagellum suggesting that they may interact and are able to, perhaps, sequester each other.

### RNAi knockdown of FPC4 is not lethal

To further characterise FPC4, we generated a PCF tetracycline-inducible, FPC4 RNAi cell line. RNAi induction decreased FPC4 mRNA levels, but total depletion was not achieved even after 10 days of induction (S2A Fig). RNAi knockdown of FPC4 in PCF had no impact on cell growth (S2B Fig) or cellular morphology, even though the protein was not detected by immunofluorescence after 48h of induction (S2C Fig). This suggests that even small amounts of protein might be sufficient for cell survival. Alternatively, other HC proteins with no predicted function, such as Tb927.4.3120, might compensate for FPC4 RNAi knockdown [34]. However, knockout attempts were unsuccessful in PCF and BSF, suggesting that FPC4 might be essential.

### Identification of the FPC targeting domain of FPC4

**Fig 5:**
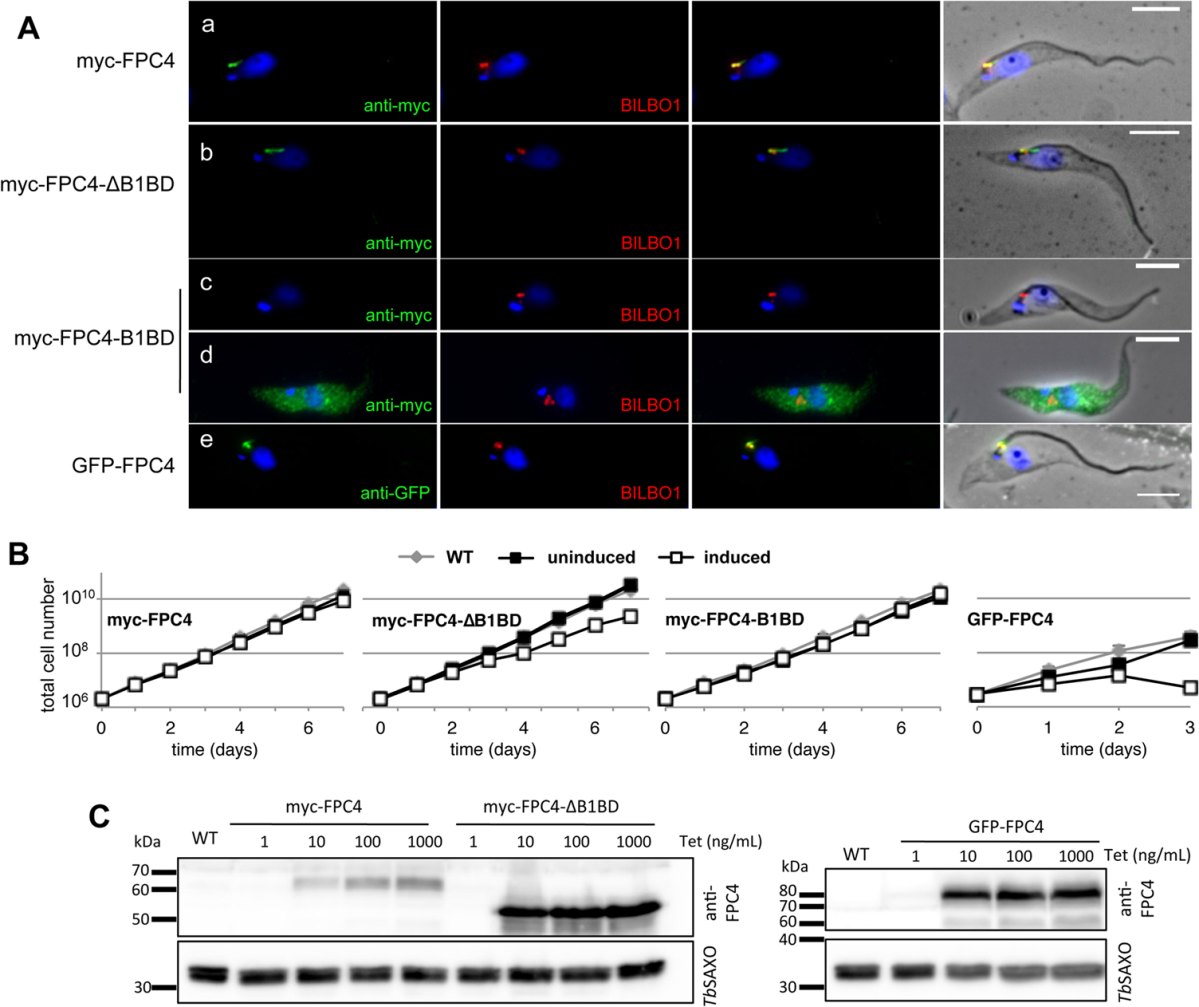
The B1BD of FPC4 is not involved in the targeting to the FPC/hook complex. (A) Immunofluorescence on cell lines over-expressing (48h of induction) myc-FPC4 (a), myc-FPC4-ΔB1BD (b), myc-FPC4-B1BD (c, d), and GFP-FPC4 (24 h of induction) (e) using anti-myc or anti-GFP (green) and anti-BILBO1 (red). Immunofluorescence was done on cytoskeletons except in (d) were cells were permeabilised. Kinetoplasts and nuclei were DAPI stained. Scale bars represent 5 μm. Initial immunofluorescence analyses were performed on two individual clones. (B) Growth curves for WT cells (grey diamond), and cells non-induced (black square) or induced (white square) for the expression of myc-FPC4, myc-FPC4-ΔB1BD, myc-FPC4-B1BD, and GFP-FPC4. Error bars represent the standard error from 3 independent experiments (errors bars are smaller than the data point mark). (C) Western blot analysis of the level of expression of myc-FPC4, myc-FPC4-ΔB1BD, and GFP-FPC4 using anti-FPC4, and *Tb*SAXO (mAb25) as loading control. Whole cell extracts from 5.10^6^ cells were loaded on 12% SDS-PAGE gels and immuno-blotted.

We generated PCF tetracycline-inducible cell lines that overexpress the N-terminal GFP tagged FPC4 or myc-tagged form of FPC4. We also made tetracycline-inducible cell lines that express an FPC4 mutant that is deleted of its B1BD (myc-FPC4-ΔB1BD) and a mutant expressing only the FPC4 B1BD (myc-FPC4-B1BD) (Fig 5A). The GFP tag did not affect FPC4 localisation because GFP-FPC4 was directed to the FPC/HC upon short time of induction (Fig 5A). Immunofluorescence labelling showed that myc-FPC4, GFP-FPC4 and myc-FPC4-ΔB1BD proteins also localised to the FPC/HC in cytoskeletons. However, myc-FPC4-B1BD was not detectable on cytoskeletons and instead was cytosolic. These data indicated that the FPC4-B1BD is neither sufficient nor required to target FPC4 to the FPC and that the domain consisting of aa 1–356 (FPC4-ΔB1BD) contains the FPC targeting sequence. Interestingly, myc-FPC4-ΔB1BD seemed to localise mostly on the shank of the HC compared to myc-FPC4. Indeed, a clear hook and shank localisation could be observed in 75% of the cells expressing myc-FPC4 (n = 50), whilst the labelling of the hook section could be observed in only 17% of the cells expressing myc-FPC4-ΔB1BD (n = 67). Also, the myc labelling on the shank was longer in cells expressing myc-FPC4-ΔB1BD (3.32 ± 0.15 μm) compared to cells expressing myc-FPC4 (1,87 ± 0.07 μm) (n at least 200 cells).

### Phenotypic analysis of FPC4 over-expression cell lines

*In vivo*, over-expression of a protein, or a mutant polypeptide that disrupts the activity of the wild-type protein, can induce dominant-negative phenotypes. Indeed, over-expression of several BILBO1 mutant proteins induced BILBO1^*RNAi*^-like phenotypes [44]. Because FPC4 RNAi knockdown did not provide information about FPC4 function, we decided to analyse the localisation of two N-ter-tagged-FPC4 (GFP and myc), and to analyse any possible phenotypes induced by long-term expression. GFP or myc tags were both used to confirm that the cell expressing either of these tagged proteins did not show modified targeting to the FPC. Indeed, neither tag affected FPC4 localisation (Fig 5A). However, expression of GFP tagged FPC4 induced cell death 3 days post induction (Fig 5B). The induction of the expression of myc-FPC4 or myc-FPC4-B1BD did not affect cell growth, whilst growth rate was reduced during expression of myc-FPC4-ΔB1BD (Fig 5B). Because of these differences in growth rates, and the fact that FPC4 could only be detected by western blot when tagged with 10TY or overexpressed, we measured the expression levels and integrity of the recombinant proteins by western blot using rat anti-FPC4 antibody. Quantification of expression levels showed that myc-FPC4-ΔB1BD and GFP-FPC4 were expressed 4x fold more than myc-FPC4 (Fig 5C). We acknowledge that the phenotypes observed (slower growth for myc-FPC4-ΔB1BD and lethality for GFP-FPC4) might be due either to the dominant negative effect of the over-expression of the proteins *per se*, or by the absence of the B1BD, or in the latter case, the presence of the GFP tag. It was, however, informative to study in more detail the phenotypes induced by the over-expression of GFP-FPC4 and myc-FPC4-ΔB1BD.

**Fig 6:**
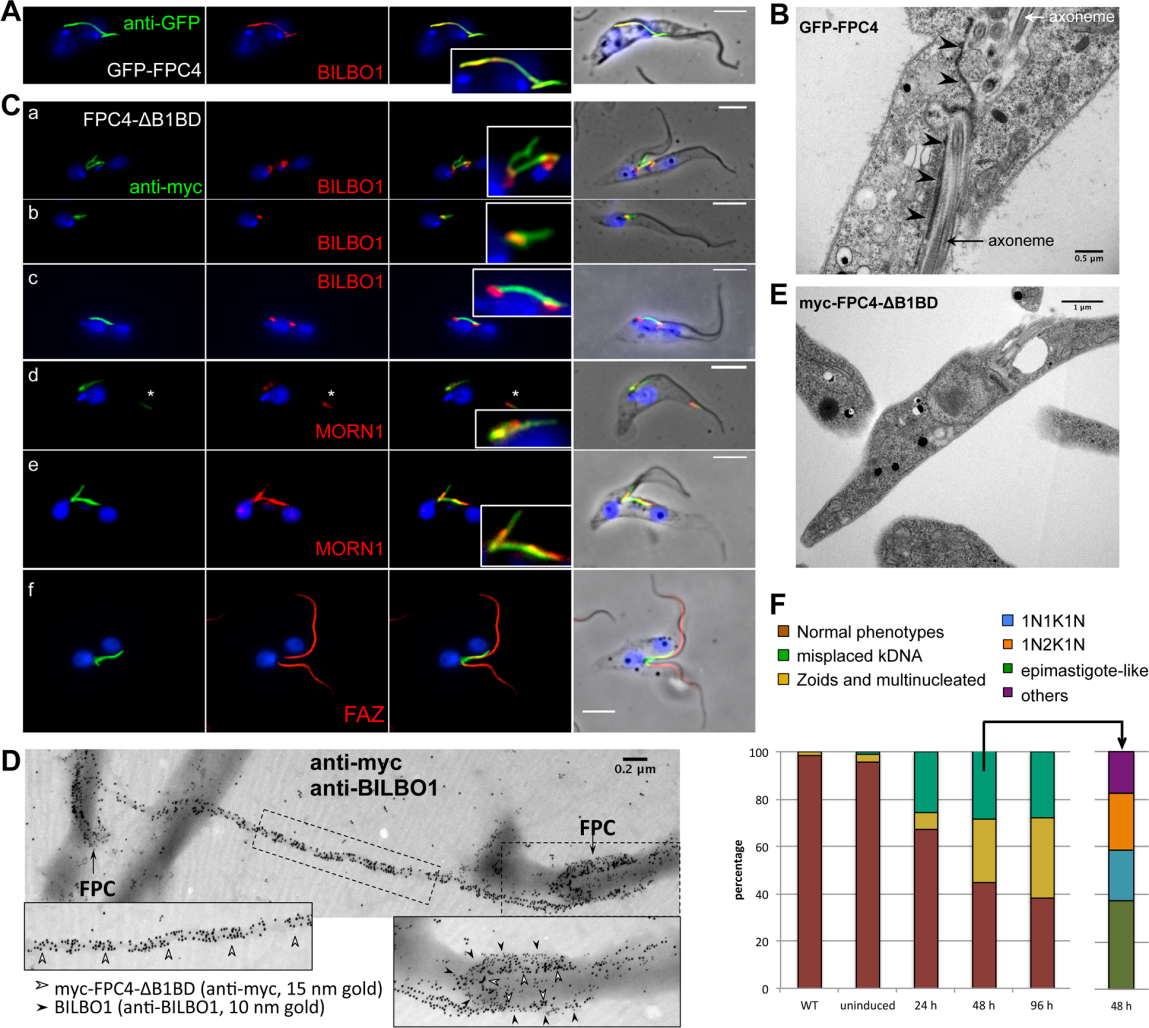
The overexpression of FPC4-ΔB1BD induced morphological phenotypes. (A) Co-localisation of GFP-FPC4 (anti-GFP, green) and BILBO1 (anti-BILBO1, red) by immunofluorescence on cytoskeletons. The cells were induced to express GFP-FPC4 for 48 h. (B) Transmission electron micrograph of a thin section of embedded cells that were over-expressing GFP-FPC4 (72 h of induction). Black arrowheads highlight the electron dense structure observed in these cells resembling the filament observed by immunofluorescence. (C) Co-localisation of myc-FPC4-ΔB1BD (anti-myc, green) and BILBO1 (red), MORN1 (red) and FAZ (red) by immunofluorescence on cytoskeletons. The cells were induced for 48 h. Several phenotypes were observed: filament connection between 2 FPCs (a, c, e), epimastigote-like cells (b), and localisation of myc-FPC4-ΔB1BD together with MORN1 at the distal end of the cell body (d). Abnormal cells still exhibit FAZ structures that were positively labelled with L3B2 (f). (D) Immuno-electron microscopy localisation of gold particles of myc-FPC4-ΔB1BD (anti-myc, 15 nm gold) and BILBO1 (anti-BILBO1, 10 nm gold) on isolated flagella from cells expressing myc-FPC4-ΔB1BD. (E) Transmission electron microscopy of thin sections of embedded cells over-expressing myc-FPC4-ΔB1BD showing an epimastigote-like phenotype. (F) Counts of cells showing different phenotypes related to kinetoplast positioning. Normal phenotype (brown - 1K1N, 2K1N, and 2K2N cells where cell morphology was normal), abnormal (yellow—Zoids and multinucleated), and abnormal (green—misplaced kinetoplast) for WT and non-induced and induced (24, 48, and 96 h) myc-FPC4-ΔB1BD over-expressing cells. The misplaced kinetoplast category was then divided into subcategories (48 h induction). Kinetoplasts and nuclei were DAPI stained and scale bars represent 5 μm in A and C. Initial immuno-localisations were performed on two individual clones.

Long induction (72h) of GFP-FPC4 induced the formation of a long GFP-positive filament, within cells, that connected two FPCs together (Fig 6A). Electron microscopy of thin sections of such cells confirmed the presence of an unusual electron dense fibre associated with the FPC (Fig 6B). This filament was positively decorated when probed with anti-BILBO1 by immunofluorescence (Fig 6A). Over-expression of myc-FPC4-ΔB1BD produced cells where 7.5% (±0.43) of the new kinetoplasts were positioned between the two nuclei (NKKN configuration) instead of a proximal position relative to the new nucleus (KNKN), without affecting cell body length. FAZ labelling appeared normal in these cells (Fig 5C). A filament was also observed between both old and new FPC in these cells (Fig 6C).

Electron micrographs of thin sections from the GFP-FPC4 induced cell lines illustrated the presence of unidentified material in the FP suggesting an alteration in the structure, function or integrity of the structure (Fig 6B). Contrary to the GFP-FPC4 cell line, over-expression of myc-FPC4-ΔB1BD did not induce these phenotypes or cell death (Fig 6C). However, phenotypic similarities were observed; for example, (a) a fibre connecting the two FPCs, and (b) formation of epimastigote-like cells. We occasionally observed the localisation of myc-FPC4-ΔB1BD together with MORN1 at the distal anterior end of the cell body and adjacent to the flagellum (Fig 6C, asterisk in d). Further, a connection between two FPCs was clearly observed by electron microscopy on isolated flagella (Fig 6D). Double immuno-gold labelling of isolated flagella from myc-FPC4-ΔB1BD cells illustrates that both FPCs are decorated with anti-myc and anti-BILBO1 gold, but the connecting fibre was only decorated with anti-myc.

Examples of a myc-FPC4-ΔB1BD expressing cell with a mispositioned kinetoplast are shown by IF and EM (Fig 6Cb and 6E). This mis-positioning was observed in 1K1N, 2K1N and 2K2N cells with different configurations. The different categories of misplaced kinetoplast containing cells represented 30% of the total cell population after 48h of induction (n = 3 with at least 400 cells). These categories were epimastigote-like (37%), 1N1K1N (21%), 1N2K1N (24%), and 1K1N1K, 1K2N, 1K2N1K, KKNN grouped as others (17%) (Fig 6F). In WT 2K2N cells used in this work, the average distance between 2 kinetoplasts is 4.5 μm (± 0.2). This distance was reduced in myc-FPC4-ΔB1BD to 3.2 μm (± 0.3) (n = 3, at least fifty 2K2N cells), with only a very slight cell body length reduction, if any (cell body length was 20.2 μm (± 0.3) in WT, and 19.3 μm (± 0.5) in myc-FPC4-ΔB1BD).

### FPC4 is a microtubule binding protein

**Fig 7:**
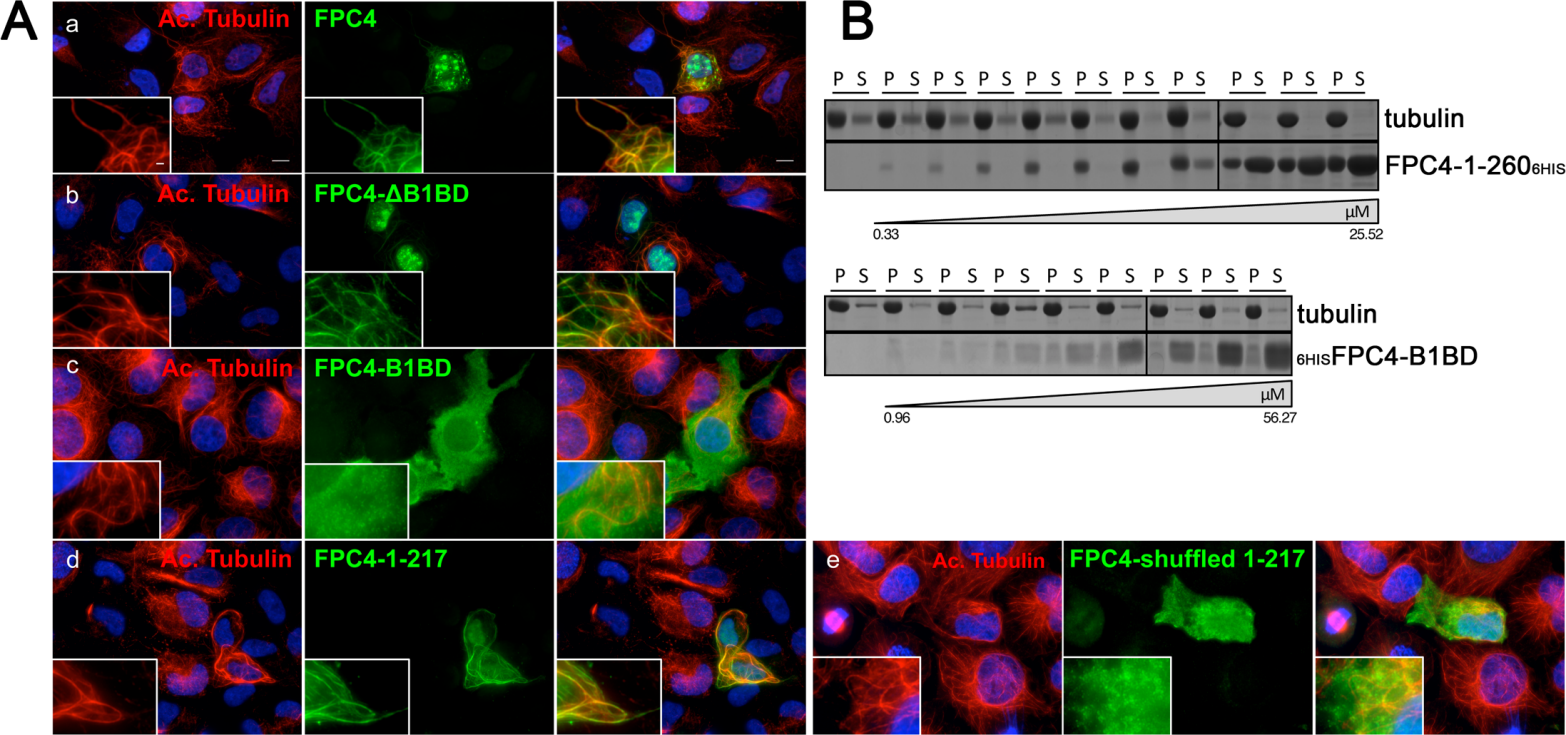
FPC4 is a microtubule binding protein. (A) Anti-acetylated tubulin and anti-GFP immunolabelling on U-2 OS cells expressing GFP tagged FPC4 (a), FPC4-ΔB1BD (aa 1–356) (b), FPC4-B1BD (aa 357–444) (c), FPC4-1-217 (d), and FPC4-shuffled-1-217 (e). Scale bar represents 10 μm. The transfections were performed more than three independent times. (B) Microtubule binding assay (n = 3). Increasing concentrations of purified FPC4-1-260_6His_ (a) or _6His_FPC4-B1BD were incubated with fixed concentration (7.2 μM) of tubulin (polymerised microtubules). After centrifugation, pellets (P) and supernatants (S) were loaded on SDS-PAGE (12% in a, 15% in b). Proteins were stained using Instant Blue.

When expressed in U-2 OS cells, FPC4-GFP localised on filamentous structures resembling microtubules (MTs) (Fig 1B). This MT localisation was confirmed by co-immunolabelling FPC4-GFP with acetylated tubulin (Fig 7Aa), suggesting that FPC4 is a MT-binding protein. Also, GFP recombinant FPC4-ΔB1BD and FPC-1-217 proteins co-localised with MTs, whilst FPC4-B1BD was cytoplasmic, showing that the B1BD is not involved in MT binding (Fig 7Ab, c, d). Because the basic pI of FPC4 might induce artifactual MT binding [49], and that FPC4-1-217 domain is basic (pI 11), its primary sequence was shuffled (the amino acid composition remains the same, but their sequence within the protein is different, S3 Fig). Shuffled-FPC4-1-217 was not able to co-localise with MT and was cytoplasmic, demonstrating the MT-binding specificity of FPC4 (Fig 7Ae).

To test FPC4 microtubule binding *in vitro*, we expressed and purified the B1BD domain of FPC4 (_6His_FPC4-357-440) and FPC4-1-260_6His_ from *E*. *coli*. Both recombinant proteins were soluble and could be used in a MT binding assay (Fig 7B). Increasing concentration of FPC4-1-260_6His_ (0.33 μM to 25.52 μM) and of _6His_FPC4-357-440 (0.96 μM to 56.27 μM) were mixed with fixed concentration of polymerised tubulin (7.2 μM). After centrifugation (16,100 g at 22°C), supernatants and pellets were loaded on SDS-PAGE and the proteins stained with Instant Blue. The presence of the protein of interest in the pellets, bound to tubulin, demonstrates the MT-binding property of the protein. This was the case for FPC4-1-260_6His_, which was found in the pellets up to a saturating concentration (5.6 μM); above this concentration, the protein was also observed in the supernatant. On the contrary, _6His_FPC4-357-440 was found in the supernatants and thus did not bind to the MTs (Fig 7B).

### FPC4 binds to the remnant MtQ complex of isolated flagella

**Fig 8:**
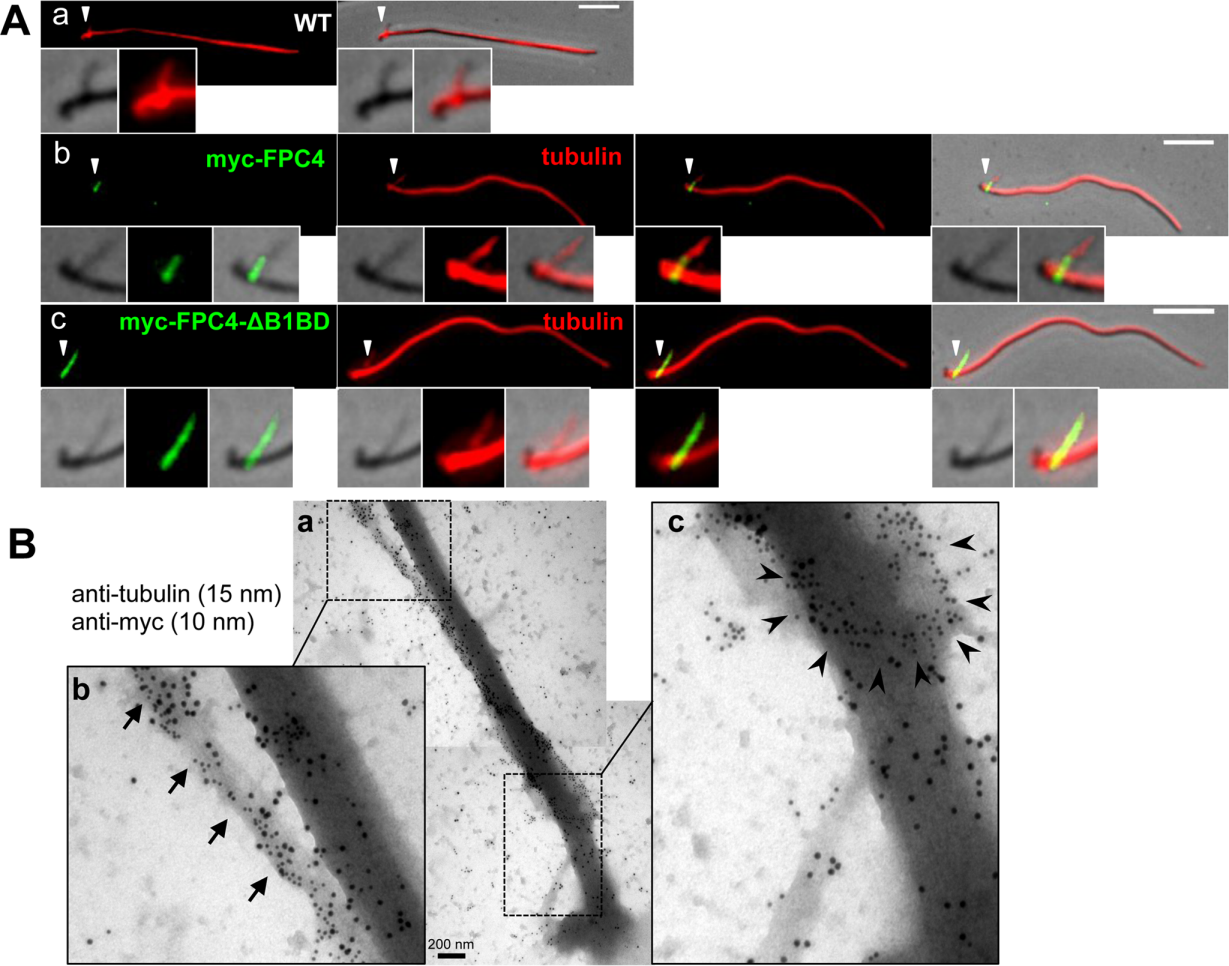
FPC4 binds to the remnant MtQ complex of isolated flagella. (A) Co-labelling of myc-tagged proteins (green) and tubulin (red) on isolated flagella from WT cells (a), and cells over-expressing myc-FPC4 (b) or myc-FPC4-ΔB1BD (c). White arrowheads are pointing to the zoom areas. Scale bars 5 μm. (B) Immuno-gold labelling of myc-FPC4-ΔB1BD (anti-myc, 10 nm gold beads) and tubulin (15 nm gold beads) on isolated flagella. In the zoom image (b), the arrows highlight the MtQ decorated with anti-tubulin and anti-myc labelling that appears close to the MtQ. In the zoom image (c), the black arrowheads highlight the FPC structure decorated by the anti-myc antibody. All the experiments were performed more than three independent times.

FPC4 is a MT-binding protein *in vitro* and in U-2 OS cells but does it localise to MT containing structures in trypanosomes? In PCF *T*. *brucei* isolated flagella, the MtQ is partially retained and can be decorated with anti-α-tubulin where it is observed as a short structure (named here the MtQ complex) close to the BBs (Fig 8A). As expected from the localisation in CK, myc-FPC4 and myc-FPC4-ΔB1BD localised to the proximal end of the isolated flagellum close to the BBs. Moreover, the remnant MtQ complex was partially decorated with myc-FPC4 and fully decorated with myc-FPC4-ΔB1BD (Fig 8A). The co-localisation on the MtQ complex of myc-FPC4-ΔB1BD and tubulin was confirmed by immuno-gold electron microscopy on isolated flagella (Fig 8B). The axoneme and the MtQ were decorated with anti-α-tubulin. The FPC and the MtQ, from the proximal to the distal end, were decorated with anti-myc, but the axoneme was negative for myc label.

Considering that FPC4 is a MT-binding protein (*in vitro* and in the U-2 OS cells), our data imply an interaction between FPC4 and the MtQ, especially since it co-localises with MORN1 and MORN1 localises to the MtQ within the shank of the HC [43].

Taken together, our data demonstrate that FPC4 is a BILBO1 binding protein that localises at the FPC and the HC. Moreover, FPC4 is a MT-binding protein that may associate with the MtQ. This implies that FPC4 has multiple binding partners and is a protein involved in linking the FPC, HC and possibly the MtQ.

## Discussion

The flagellar pocket collar (FPC) and the hook complex (HC) are two essential *T*. *brucei* cytoskeletal structures whose molecular components are inadequately defined [29,50]. Furthermore, the molecular processes involved in their segregation during the cell cycle are poorly understood. Although recent data identified the Polo-like kinase *Tb*PLK as being involved in HC duplication, BB segregation, and flagellum attachment [51,5,32,52], BILBO1 remains the only FPC protein to be identified and characterised.

As stated above, BILBO1 is the only FPC protein to be described, whereas MORN1, TBCCD1 and LRRP1 are the HC proteins that have been characterised in detail [25,33,39,41,42]. Following up on results showing that BILBO1 is a structural protein in *T*. *brucei* [46,44], we hypothesize that it is unlikely that BILBO1 is the only protein required for the biogenesis and function of the FPC. For this reason, we screened for BILBO1 interacting partners using a yeast two-hybrid genomic library and FPC4 was identified.

In this study, we demonstrate that FPC4 is a MT-binding protein that localises together with MORN1 at the HC and is sandwiched between the HC and the FPC. The interface between the HC and the FPC has FPC4 labelling and logically this is where FPC4 interacts with BILBO1. Also, FPC4 co-localises with MORN1, which has been shown by immuno-gold labelling to be adjacent to and on the MtQ [43]. Our data imply that FPC4 also localises close or onto the MtQ and provide the first evidence of a possible molecular link between these structures. The precise role of the FPC4 MT binding is still unclear, but one possible hypothesis is that FPC4 connects components of the FPC and of the HC to the MtQ. FPC4 could have MT stabilization function (as suggested by the presence of FPC4 on acetylated MTs in U-2 OS cells).

*In vitro* and *in vivo* analysis demonstrated that FPC4 interacts with the BILBO1 NTD *via* its C-terminal domain (B1BD) in a 1:1 ratio. BILBO1 NTD is neither required for targeting to the FPC nor for polymerization [45,44]. However, it folds into an ubiquitin-like structure most likely playing a role in protein interaction [45]. Here, we have tested two new sets of mutations Centre-Mut and Edge-Mut that both localise at the FPC similar to mut1 and mut2 (45), and demonstrated the critical role of Y87 and F89 residues (Centre-Mut) in the interaction with the FPC4-B1BD. Centre-Mut over-expression in *T*. *brucei* leads to rapid cell death, and *in vitro*, Centre-Mut completely abolishes the interaction with FPC4. This demonstrates a direct impact on the interaction of these two residues, which can therefore be used in a drug design or drug-screening project. The different effects in the two mutants, Centre-Mut and Edge-Mut, might attribute to their specific locations at the FPC4-binding site and different contributions to FPC4 binding. The Centre-Mut is located at the centre of the binding pocket and consists of two aromatic residues (Y87 & F89), which likely provide a strong hydrophobic interaction between the two proteins. The Edge-Mut is at the edge of the pocket and contains two charged residues (K60 & K62), which may provide only a peripheral contact mediated by a much weaker electrostatic interaction. BILBO1 NTD is not required for FPC4 targeting, and overexpression of the BILBO1 Centre-Mut mutant, which cannot interact with FPC4, is lethal. This suggests that the mutation may prevent proper binding of other partner proteins and, in consequence, is affecting the function of the FPC.

Reduction of FPC4 expression by RNAi knockdown did not affect cell growth or cell morphology similar to the result reported in the genome-wide RNA interference target sequencing analysis by Alsford and colleagues [53]. However, knockout attempts were unsuccessful in PCF and BSF, suggesting that FPC4 might be essential. Further, over-expressing FPC4 (GFP-FPC4 or FPC4-ΔB1BD) led to the formation of a single fibre that formed between the old and the new FPC and generated epimastigote-like cells, presumably because this fibre impinges on correct FPC segregation. Several studies have shown that limiting the kinetoplast segregation (or the FPC segregation) leads to misplaced kinetoplasts and epimastigote-like cells [54–56]. It was previously reported that transition to epimastigote-like cells can be the result of the knockdown of FAZ proteins [15,56]. Indeed, FPC4 has been recently identified by proximity-dependent biotin identification (BioID) as a *Tb*SAS-4 near neighbour [37]. *Tb*SAS-4 is concentrated at the distal tip of the FAZ filament and is involved in regulating FAZ length. FPC4 and *Tb*SAS-4 might, therefore, come in close proximity when the new FAZ elongation is initiated. However, FAZ was not obviously affected in the FPC4 over-expressing cell lines, as its immunolabelling appeared normal. The epimastigote-like cells most probably come from a downstream effect of the delayed segregation of FPC—HC due to the connecting fibre between the two FPCs. Interestingly, this fibre contains not only FPC4 but also MORN1 (S4 Fig), and BILBO1 (in the case of expression of GFP-FPC4, but not in the case of the expression FPC4 deleted of its B1BD), suggesting that FPC4 can sequester BILBO1 under these conditions and also supports the data from the FPC4-BILBO1 binding assays. Because FPC4 binds MT, and the MtQ is not apparent when the fibre is formed, we also questioned whether this fibre is microtubule-based. The fibre has an average width of 56 nm (n = 33) and could, theoretically, accommodate one or two microtubules (25 nm in diameter). As FPC4 is present on or close to the MtQ, this fibre could be an extension (or a modification) of a subset of the MtQ microtubules. However, in our hands, myc and α-tubulin co-immuno-labelling and visualisation of the fibre using electron microscopy was not conclusive, perhaps due to limited access of anti-tubulin antibodies to the fibre. Alternatively, FPC4 or MORN1 could form polymers in the absence of microtubules as previously suggested for MORN1 that was immuno-gold-labelled on the tendril, an uncharacterised filament, devoid of tubulin, that seems to run parallel to the MtQ between the BBs and the FPC [43]. Interestingly, RNAi knockdown of Spef1, a MtQ-binding protein that localises between the BB and the FPC, leads to the inhibition of the assembly of a new MtQ. Spef1 knockdown also prevented FPC and HC segregation, and resulted in unsegregated and misplaced kinetoplasts [55]. Thus, over-expressing the microtubule-binding domain of FPC4 (GFP-FPC4 or FPC4-ΔB1BD) could stabilise the MtQ and prevent FPC separation.

The potential association between FPC4 and the MtQ is restricted to a specific region of the MtQ (the FPC and along the shank of the HC). Considering that FPC4 binds to acetylated tubulin in U-2 OS cells (Fig 7), it could imply that the MtQ tubulin is also post-translationally modified. Unfortunately, MtQ-specific modifications have not been identified so far, although IFA images of flagella probed with anti-acetylated tubulin antibody suggest that the MtQ is acetylated [57]. The restricted MtQ localisation of FPC4 could arise also *via* the interaction with BILBO1 and MORN1 and other proteins that would retain FPC4 to slide along the MtQ. This is supported by the fact that deletion of the BILBO1-binding domain allows FPC4 to localise to the anterior end of the cell body as observed in Fig 5, suggesting that the protein was able to be associated with the MtQ when released from the interaction with BILBO1.

FPC4 might bind or sequester MORN1 as suggested by the presence of MORN1 on the fibre connecting the two FPCs and at the anterior end of the cell body (most likely at the distal tip of the MtQ/FAZ), as well as on the structure observed in the flagellum of BILBO1^*RNAi*^ cells. It is possible that under these over-expression conditions, either FPC4 deleted of its B1BD is free to move along the MtQ or other MTs and can accumulate at the tip of the MtQ, or that the binding sites at the FPC/HC are saturated and the excess protein is also to along the MtQ where it accumulates.

The reasons for FPC4 localisation on the detached flagellum of BILBO1^*RNAi*^ cells is unclear, but can be explained by its affinity for MTs. An alternative explanation is related to Centrin2. Centrin2 localises to the BBs, the bilobe, and the flagellum [41,58]. Since Centrin2 is also part of the bilobe, in induced BILBO1^*RNAi*^ cells some components of the bilobe could be assembled together and be targeted to the flagellum in absence of a real bilobe structure.

Despite not having any homology at the primary sequence level, the folding of BILBO1 NTD is similar to the N-terminal PB1 domain of Par6 [59]. Par6 is part of the Par complex, a membrane associated complex of polarity proteins, consisting primarily of Par6, Par3 and aPKC (atypical Protein Kinase C). This complex regulates cellular processes involved in epithelial cell polarity or tight junction formation in epithelial cells [60,61]. Par3 is phosphorylated by aPKC and binds to MTs. Par3 and Par6 are multi-modal scaffold proteins that bind to each other as well as to other proteins. [62]. The analogy between this Par complex and BILBO1 –FPC4 –MtQ complex is tempting, especially when considering *Tb*PLK, the unique Polo-like kinase homologue in *T*. *brucei*, a key player in the initiation of the cell cycle. *Tb*PLK RNAi leads to a BB segregation defect most probably because it causes defects in FPC duplication or segregation [36,63,51,5,32,54]. Further, phosphoproteomics and BioID approaches to identify *Tb*PLK binding partners and substrates identified several bilobe proteins, but also the uncharacterised FPC protein Tb927.11.5640 [36]. Thus, FPC4 interaction with one or several partners might be regulated by phosphorylation.

We also localised FPC4 on BSF cytoskeletons and also observed similar FPC and HC localisation as described for PCF. However, we also observed in BSF some labelling towards the BB, similar to the region where *Tb*Spef1 label is located [55]. This would suggest higher levels of FPC4 expression in BSF, easier access for the antibody, or higher rate of traffic to the FPC. Interestingly, in Xenopus embryos, Spef1 (also named CLAMP) interacts with aPKC and stabilizes MT [64]. Further work on the role of FPC4 might help understanding how the FPC is segregated and if the MtQ is involved in this process.

In summary, our results demonstrate that FPC4 is a multi-partner protein that is involved in FPC segregation and is the first identified link between the FPC, the HC and possibly the MtQ. The dual role of FPC4, as BILBO1 partner and as a microtubule binding protein, highlights the importance of BILBO1, making it not only a real ring of power for *T*. *brucei* but also a potential target for therapeutic intervention. The resolution of the 3D structure of the BILBO1-NTD/FPC4-B1BD complex is on-going, and it is planned to identify in detail the key amino acid residues in FPC4 that are involved in this interaction. The FPC (as characterised by BILBO1) is an essential cytoskeletal structure, which is also present in *T*. *cruzi* and *Leishmania* ssp [65,66] and BILBO1 is highly conserved [25,45]. Thus, the FPC is a generic target in kinetoplastids as well as an important component in understanding cytoskeleton biogenesis.

## Materials and methods

### Cell lines, growth conditions and transfection

Genomic DNA of *T*. *brucei* TREU927/4 GUTat10.1 [67] was used to amplify by PCR the FPC4 ORF (*Tb*927.8.6370). The work described in this study used the PCF *T*. *brucei* 427 29–13 and BSF 427 90–13 strains co-expressing the T7 RNA polymerase and the tetracycline repressor [68,69] (named WT throughout the manuscript otherwise stated). PCF cells were cultured at 27°C in SDM79 medium (Sigma) containing 10% (v/v) heat-inactivated foetal calf serum, 10 μg.ml^-1^ Hemin, hygromycin 25 μg.mL^-1^, and neomycin 10 μg.ml^-1^. Cells were transfected as described in [69] with the transfection buffer described in [70]. We generated the SmOxP427 cell line expressing endogenously tagged 10xTY1-FPC4 using the pPOTv4-10xTY1 vector for PCR template as described in [71]. After transfection, the cells were selected using blasticidin at 20 μg.mL^-1^. BSF *Tb*427 90–13 MITat 1.2 (named in this study as BSF wild-type, WT) were cultured at 37°C as described in [72] in IMDM medium containing 10% (v/v) heat-inactivated foetal calf serum, 36 mM sodium bicarbonate, 136 μg.mL^-1^hypoxanthine, 39 μg.mL^-1^ thymidine, 110 μg.mL^-1^ sodium pyruvate, 28 μg.mL^-1^ bathocuproine, 0.25 mM β-mercaptoethanol, 2 mM L-cysteine, 62.5 μg.mL^-^1 kanamycin, 2.5 μg.mL^-^1 neomycin, and 5 μg.mL^-^1 hygromycin. They were transfected using the AMAXA electroporator (Lonza) as described in [70]. Clones were selected after serial dilutions. Over-expression and RNAi were induced with tetracycline at 10 μg.mL^-1^ for 48 h otherwise stated. U-2 OS cells (human bone osteosarcoma epithelial cells, ATCC Number: HTB-96 [73] were grown and transfected as described in [44] and processed for immuno-fluorescence 24h post-transfection.

### Plasmid construction

#### E. coli expression vectors

To produce the anti-FPC4 rat polyclonal antibody, the FPC4 ORF was amplified from *T*. *brucei* 927 genomic DNA and cloned into pET28a+ (Novagen) in frame with the N-terminal 6-histidine-tag and the C-terminus 6-histidine-tag to produce the _6His_FPC4_6His_. FPC4-1-260_6His_ and FPC4-_6His_357-440 were cloned into pET28a+ (Novagen) in frame with a C-terminal 6-histidine-tag and an N-terminus 6-histidine-tag respectively. *Tb*BILBO1-NTD’ (aa1-120) was cloned into pET-15b (Novagen) between NdeI and BamHI sites, which provides an N-terminal 6-Histidine tag cleavable by thrombin. *Tb*FPC4-B1BD (aa357-440) was cloned into a custom vector MalpET that adds an N-terminal MBP-10×His tag, cleavable by the tobacco etch virus (TEV) protease.

#### Trypanosome vectors

For the RNAi experiments in PCF, the *FPC4* fragment (bp 164–754) was cloned as a stem-loop construct between the *Hind*III/*BamH*I restriction sites of the pLew100 vector [69]. The full-length *FPC4* ORF was cloned into the *HpaI-XbaI* sites of pLew100GFPX vector containing a N-terminal GFP-tag to express GFP-FPC4 (vector modified from [74]), and between *BamH*I and *Hind*III sites of pJM-2 (pLEW100 based plasmid, a kind Gift from A. Schneider) to express N-terminal 3xmyc tag FPC4 and FPC4 truncations. For C-terminal 3xmyc endogenous tagging in PCF, long primers were designed as described in [75] and the PCR was performed with pMOT23M vector as template. The PCR product was directly used for the transfection as described in [75]. Ty1-tagged Centre-Mut and Edge-Mut BILBO1 were cloned as described in [45].

#### Yeast two-hybrid vectors

Genes encoding proteins and truncations were amplified and cloned into the *Sfi*I-*BamH*I sites (for *FPC4*) and *EcoR*I-*BamH*I (for *BILBO1*: *Tb*927.11.12150) of the pGADT7 and pGBKT7 plasmids (Clontech).

#### Mammalian expression vectors

The BILBO1 ORF was cloned into the pcDNA3 vector as described in [44], from which Edge-Mut and Centre-Mut were generated using the QuickChange II site-directed mutagenesis kit (Agilent). The FPC4, FPC4-B1BD, FPC4-ΔB1BD, and shuffled FPC4-1-217 ORFs were cloned into pcDNA3.1 CT-GFP TOPO (Invitrogen) in frame with a C-terminal GFP tag. Shuffled FPC4-1-217 DNA sequence was synthesised by Eurofins (Germany).

### Semi-quantitative RT-PCR

Semi-quantitative RT-PCR was performed on total RNA with a primer pair that amplified a (673 bp) fragment of FPC4. RNA 18S was used as control for RNA integrity and for loading and was amplified with a primer pair amplifying a 181 bp fragment [76]. Briefly, total RNA of 10^8^ trypanosome cells was collected and resuspended in 500 μL of TRIzol RNA (5PRIME) and treated according to the manufacturer’s instructions. Collected RNA was then treated with DNase-TURBO (Ambion) for 30 minutes at 37°C. 100 ng of RNA were used for the RT-PCR reaction *via* SuperScript III One-Step RT-PCR System with Platinum *Taq* DNA polymerase (Invitrogen) according to the manufacturer’s instructions.

### Protein purification

BL21 (*DE3*) (Novagen) bacterial strain was transformed with pHis-FPC4-His, pFPC4-1-260_6His_, pFPC4-_6His_357-440 and grown in 200 mL of LB + kanamycin (50 mg.mL^-1^) to OD_600 nm_ of 0.5. Expression of the recombinant proteins was induced by adding 1 mM isopropyl-beta-D-thiogalactopyranoside (IPTG) for 2 h at 37°C. Cells were harvested and resuspended in 20 mL of NaPi 50mM pH 8.0 or 9.0, NaCl (500 mM for pHis-FPC4-His, 250 mM for pFPC4-1-260-His and pFPC4-His-357-440) (buffer A) with protease inhibitors (Calbiochem Cocktail III) and lysozyme (1mg.mL^-1^). Cells were lysed by sonication. Inclusion bodies (pHis-FPC4-His) were pelleted by centrifugation (10,000 g) for 30 min 4°C and resuspended in 20 mL of buffer A, sonicated and washed twice in buffer A. The pellet was solubilised in buffer B (buffer A plus 8 M Urea, pH 8.0). The _6His_FPC4_6His_ protein was purified by affinity chromatography on a 1.25 mL HIS-Select Cartridge (Sigma) Nickel affinity gel and eluted with a gradient of Imidazole (0 mM—250 mM) in buffer B. The protein was dialysed against buffer A plus 6 M urea at 4°C, and used to immunise rats for antibody production (Eurogentec). FPC4-1-260_6His_ and FPC4-_6His_357-440 were present in the supernatant after sonication (in NaPi 50mM pH8 or pH9, 250 mM NaCl) and were purified on affinity columns HisTrap FF (1ml) and eluted with a 20-500mM gradient of imidazole. Proteins were dialysed in PEMD (100mM PIPES-NaOH pH6.8, 1mM EGTA, 1mM MgCl_2_, 1mM DTT). Protein concentration was assessed with the Pierce 660nm protein assay kit.

For SLS and ITC experiments, transformed bacterial cells were grown at 37°C until OD_600nm_ reached 0.6–0.8, and then subjected to cold shock on ice for 10 min. Afterwards, the cell culture was further incubated at 16°C for 30 min before 0.5 mM of IPTG was added to the cell culture to induce expression. The cells were harvested the next day by centrifugation in a Sorvall GS-3 rotor (6,000 g, 12 min, 4°C) and resuspended in cold lysis buffer (20 mM Tris-HCl (pH 8.0), 300 mM NaCl, 20 mM imidazole, and 5% (v/v) glycerol). The cells were broken by the EmulsiFlex-C3 homogenizer (Avestin) and the lysate was cleared by centrifugation at 30,000 × g for 30 min. The supernatant was filtered through a 0.4-μm filter and loaded onto a Ni-HiTrap column (GE Healthcare) pre-equilibrated in the same lysis buffer. The column was washed with 5 × column volume (cv) of lysis buffer, and bound protein was eluted by a linear gradient concentration of imidazole (20–600 mM, 10 × cv) in the same lysis buffer. The fusion tags on BILBO1-NTD’ and FPC4-B1BD were removed by incubating with ~1% (w/w) of thrombin and ~2% (w/w) of TEV, respectively, overnight at 4°C. Target proteins were further purified with a Superdex-200 16/60 column (GE Healthcare) pre-equilibrated with 20 mM Tris-HCl (pH 8.0), 100 mM NaCl and 5% (v/v) glycerol. Fractions containing each target protein were pooled, concentrated, and used for subsequent analysis.

### Immuno-fluorescence

#### Wide-field fluorescence microscopy

Wide-field fluorescence microscopy on *T*. *brucei*. For cytoskeleton preparation, cells were processed and fixed as described in [44], except for BSF that were washed in vPBS (NaCl 0.8 mg.mL^-1^, KCl 0.22 mg.mL^-1^, Na_2_HPO_4_ 22.7 g.mL^-1^, KH_2_PO_4_ 4.4 mg.mL^-1^, sucrose 15.7 mg.mL^-1^, glucose 1.8 mg.mL^-1^), resuspended in 0.25% Nonidet P-40 (IGEPAL), 100 mM PIPES-NaOH pH6.9, 1 mM MgCl_2_, and loaded on poly-L-lysine-coated slides for 10 min. To prepare flagella, PCF cytoskeletons were prepared in solution (1% Nonidet P-40 (IGEPAL), 100 mM PIPES-NaOH pH6.9, 1 mM MgCl_2_) and further extracted with 1M KCl (in 1% Nonidet P-40 (IGEPAL), 100 mM PIPES-NaOH pH6.9, 1 mM MgCl_2_) for 5 min. After two washes (100 mM PIPES-NaOH pH6.9, 1 mM MgCl_2_), flagella were loaded on poly-L-lysine-coated slides. After either 4% paraformaldehyde (PFA) fixation for 5 min at RT (then 10 mM glycine neutralization) or -20°C methanol fixation for 30 min, the slides were washed in PBS twice for 5 min and incubated with primary antibodies in PBS for 1 h in a dark moist chamber—anti-BILBO1 (rabbit polyclonal raised against a purified untagged recombinant fragment of BILBO1 (aa 1 to 110) (COVALAB), 1:4,000 dilution); anti-GFP (mouse monoclonal (Molecular Probes), 1:1,000 dilution); anti-FPC4 (rat polyclonal, 1:250 dilution); anti-MORN1 (rabbit polyclonal 1340 [34], 1:4,000 dilution, a kind gift from B. Morriswood); anti-α-tubulin DM1a (mouse monoclonal (Sigma), 1:500 dilution) or TAT1 (1:50 dilution, a kind gift from K. Gull), anti-FAZ L3B2 (mouse monoclonal, neat dilution, a kind gift from K. Gull [77]), anti-myc (mouse monoclonal 9E10, a kind gift from K. Ersfeld, 1:20 dilution); anti-myc (rabbit, polyclonal (Santa Cruz), 1:1,000 dilution), anti-TY1 tag (BB2, 1:50, a kind gift from P. Bastin, [78]). Following the primary antibody incubation, samples were washed twice (5 min) in PBS and incubated 1h with the secondary antibodies–anti-rat IgG conjugated to Alexa fluor 488 (Molecular Probes, 1:400); anti-rabbit IgG conjugated to FITC (Sigma, 1:100); anti-rabbit IgG conjugated to Alexa fluor 594 (Molecular Probes, 1:100); anti-mouse IgG conjugated to FITC (Sigma, 1:100); anti-mouse IgG conjugated to Alexa fluor 647 (Molecular Probes, 1:100)–according to the primary antibody combination. After two 5 min washes in PBS, kinetoplasts and nuclei were labelled for 5 min with DAPI (10 μg.mL^-1^) followed by two PBS washes. Slides were mounted with SlowFade® Gold Kit (Molecular Probes, S-36936). Images were acquired with Metamorph software, on a Zeiss Imager Z1 or Axioplan 2 microscope, using a Photometrics Coolsnap HQ2 camera, at 100x or 63x (NA 1.4) and processed with ImageJ.

Wide-field fluorescence microscopy on U-2 OS cells. U-2 OS cells grown on glass coverslips were washed with PBS and fixed in 3% PFA and permeabilised or extracted for 2 min with an extraction buffer (0.5% TX-100, 10% glycerol in EMT [60 mM PIPES-NaOH pH6.9, 25 mM HEPES, 10 mM EGTA, 10 mM MgCl_2_]) then fixed in 3% paraformaldehyde in PBS for 10 minutes (at RT) then processed for immunofluorescence as in [44]. The primary antibodies–anti-BILBO1 1–110 (rabbit polyclonal, 1:4000 dilution); anti-acetylated-tubulin (mouse monoclonal (Sigma), 1:1,000 dilution), anti-living colour (rabbit polyclonal (Clontech), 1:1,000 dilution) and anti-GFP (mouse monoclonal (Molecular Probes), 1:1,000 dilution)–were incubated for 1h in a dark moist chamber. After two PBS washes, cells were incubated for 1h with the secondary antibodies–anti-rabbit IgG conjugated to FITC (Sigma, 1:400); anti-rabbit IgG conjugated to Alexa fluor 594 (Molecular Probes, 1:400); anti-mouse IgG conjugated to FITC (Sigma, 1:400); anti-mouse IgG conjugated to Alexa fluor 647 (Molecular Probes, 1:400). The nuclei were stained with DAPI (0.25 μg.mL-1 in PBS for 5 min), then washed and mounted with Prolong (Molecular Probes S-36930). Images were acquired on a Zeiss Imager Z1 microscope with Zeiss 100x or 63x objectives (NA 1.4), using a Photometrics Coolsnap HQ2 camera and Metamorph software (Molecular Devices), and processed with ImageJ.

#### STimulated Emission Depletion (STED) confocal microscopy on *T*. *brucei*

Cells were loaded on poly-L-lysine coated round coverslips, extracted and fixed in methanol at -20°C as described in the wide-field microscopy section except with the following modifications. BILBO1 was labelled with HiTrap IgM purified anti-BILBO1 5F2B3 monoclonal antibody [25] (1:20) followed by anti-mouse Oregon green conjugated secondary antibody (Thermo Fisher scientific #O-11033). In a second step, MORN1 and 10xTY1-FPC4 were respectively labelled with rabbit polyclonal anti-MORN1 (1340) (1:4,000) and HiTrap Protein G affinity purified anti-TY1 BB2 monoclonal antibody (1:500) followed by the anti-rabbit ATTO 647N (Sigma #40839) and anti-mouse IgG1 Alexa Fluor 594 (Thermo Fischer Scientific A21125) conjugated antibodies at 1:100 dilution in PBS. Coverslips were mounted on glass slides using Prolong Gold anti-fading reagent (Thermo Fisher Scientific #P36930). Images were acquired on a Leica DMI6000 TCS SP8 X-STED microscope with a 100x objective (NA 1.4), de-convolved with Huygens Pro 16.10, and 3D reconstructions were generated using Imaris X64 8.1.2.

### Yeast two-hybrid assay

*FPC4* and *BILBO1* ORFs were cloned into pGADT7-AD and pGBKT7 respectively. The pGADT7-AD (prey) and pGBKT7 (bait) based plasmids were transformed in the Y187 and Y2HGold yeast cell lines respectively. After production of diploids cells, interaction tests were done on SC-L-W-Histidine media, and control media containing histidine, using the drop test technique as described in [44].

### Microtubule co-sedimentation assays

Five mg.mL^-1^ taxol-stabilised MTs were prepared as in [79]. For co-sedimentation assays, 4 μL of MTs were mixed with different amounts of protein dialysed in PEMD buffer. Volumes were completed to 50 μL with PEMD (7.2 μM tubulin final concentration). Samples were then incubated for 1 h at 22°C and centrifuged 15 min at 16,100xg at 22°C. Supernatants and pellets were separated and brought to equal volumes in SDS sample buffer. Equal volumes of pellets and supernatants were analysed by SDS-PAGE. Gels were stained using Instant Blue (Expedeon).

### Immuno Transmission electron microscopy on isolated Flagella

Flagella were prepared as previously described in [43], loaded on Formvar/Butvar covered, charged, carbon-coated nickel grids and incubated for 10–15 min to let them adhere. The grids were then moved to a 250 μL droplet of 1% Nonidet P-40 (IGEPAL) in PEME (100 mM PIPES-NaOH pH 6.9, 1 mM MgCl2, 0.1 mM EDTA, 2 mM EGTA) and 1:10,000 complete protease inhibitor cocktail (Calbiochem) and incubated for 5 min and repeated once on a fresh droplet. The grids were then moved to a droplet containing in addition 1 M KCl, for 30 min on ice. After four washes in PEME buffer with protease inhibitors, the flagella were fixed on a 100 μL 3% PFA in PEME droplet for 5 min. PFA was then neutralised with four incubations on 100 μL droplets of 100 mM glycine in PEME. The grids were then transferred through five blocking droplets for 5 min each (0.1% BSA, 0.1% Tween-20 in PBS) then to droplets containing the primary antibodies–anti-myc (mouse monoclonal 9E10, 1:20), anti-BILBO1 1–110 (rabbit polyclonal, 1:400), anti-MORN1 1340 (rabbit polyclonal, 1:400), anti-myc (rabbit, Santa Cruz, 1:100), and anti-tubulin TAT1 (mouse monoclonal—a kind gift of K. Gull, 1:50)–and were incubated for 2h at RT in PBS 0.01% BSA and 0.1% Tween-20. The grids were washed four times in PBS 0.01% BSA and 0.1% Tween-20 and incubated with gold-conjugated secondary antibodies–EM GAR10 1:20 (anti-rabbit, 10 nm), and EM GAM15 1:50 (anti-mouse, 15 nm)–in PBS 0.01% BSA and 0.1% Tween-20 for 2h at RT. The grids were then washed twice in blocking buffer, twice in PBS and then fixed for 5 min in 2.5% glutaraldehyde in PBS. After two washes in milliQ H2O, the grids were negatively stained in 0.5% Nanovan for 5–10 sec. For the double immuno-labelling on SmOXP427 cells expressing 10xTY1-FPC4, flagella were prepared as described above, but with the following modifications. All buffers including blocking buffers contained protease inhibitors. Flagella were not fixed prior to antibody incubation. After washing, the flagella were blocked in 1% or 2% fish skin gelatin, 0.01% Tween-20 in PBS pH.7.3 for 30 min. Grids were incubated on antibody droplets sequentially for 60 min starting with mouse monoclonal anti-TY1 (BB2), diluted 1:5 in blocking buffer. Grids were then washed 3 x 5 min in blocking buffer, then incubated in 30μL droplets of anti-mouse 5nm gold (British Biocell International BBI solutions). Grids were then washed 3 x 5 min in blocking buffer and incubated in 30μL droplets of affinity purified anti-BILBO1 (mAb 5F2B3, IgM diluted 1:5 in blocking buffer) or rabbit anti-MORN1 diluted 1:250 in blocking buffer. Grids were then washed 3 x 5 min in blocking buffer and incubated on 30μL droplets of anti-mouse IgM 15 nm gold (for grids incubated with anti-BILBO1) diluted 1:10 in blocking buffer or anti-rabbit IgG 15 nm gold (for grids incubated in anti-MORN1) diluted 1:10 in blocking buffer. After incubation, grids were washed 3 x 5 min in blocking buffer, 2 x 5min in 0.1% fish skin gelatin, 0.001% Tween-20 in PBS pH 7.3, 2 x5 min PBS pH 7.3 and then fixed in 2.5% glutaraldehyde in Milli-Q water for 5 min. Samples were negatively stained with 5–10μL Aurothioglucose for 20 sec.

### EM thin section and labelling

For ultra-thin sections, a mid-log phase culture was fixed for 2h in medium with 2.5% glutaraldehyde, and consequently fixed in 2.5% glutaraldehyde in 0.1 M Sorensen’s Sodium Phosphate buffer pH 7.2. The cells were rinsed twice in milliQ H2O and incubated for 1 h in 1% OsO4 in milliQ H2O pH 7.2. After three washes in milliQ H2O, the cells were stained and fixed in 2% Uranyl-acetate (milliQ H2O) at 4°C overnight. After three 10 min washes in milliQ H2O, the cells were dehydrated in EtOH, starting from 30% EtOH, then 50%, 70% and 90% for 2h each and left in 90% EtOH overnight. The cells were incubated three times up to 1h in 100% EtOH at RT and then for 3h each with 30%, 50%, 70% and 90% EtOH: Spurs resin. Finally, the cells were incubated three times for 1h with 100% Spurs resin at RT and then overnight in 100% Spurs (Low viscosity embedding kit, EMS 14300) at RT. The resin was embedded in size “OO” BEEM capsules (EMS) and let polymerize overnight at 60°C. Thin sections were cut with Ultramicrotome LEICA EM-UCT at a thickness of approximately 70–90 nm. The sections were deposed on grids and stained with aqueous saturated uranyl acetate for 15–30 min. The grids were washes three times in boiled cooled water for 5 min each and then air-dried. An additional wash with 0.1 N NaOH for 30 sec and three washes in boiled cooled water for 5 min. Samples were visualised on a FEI Tecnai 12 electron microscope, camera ORIUS 1000 11M Pixel (resolution 3–5 nm). Images were acquired with Digitalmicrograph and processed with ImageJ.

### Western blot

Bacterial extracts, trypanosome whole cell protein lysates and purified proteins were separated on SDS-PAGE gels (10%–15%) and transferred by semi-dry (BioRad) blotting 45 min at 25V on PVDF membrane. After a 1 h blocking step in 5% Milk in PBS-0.2% Tween, the membranes were incubated with the primary antibodies diluted in blocking buffer–anti-*Tb*SAXO (mAb25 mouse monoclonal, 1:1,000 dilution [80]); anti-FPC4 (rat, 1:500 dilution) and anti-myc (rabbit polyclonal (Santa Cruz), 1:500 dilution). After three washes in blocking buffer, the membranes were incubated with the secondary antibodies–anti-mouse antibody HRP-conjugated (Jackson, 1:10,000 dilution), anti-rat antibody HRP-conjugated (Jackson, 1:10,000 dilution) and anti-rabbit antibody HRP-conjugated (Sigma, 1:10,000 dilution)–and washed twice 10 min in blocking buffer and twice 5 min in PBS. Blots were revealed using the Clarity Western ECL Substrate kit (Bio-Rad) with the ImageQuant LAS4000.

### Static light scattering (SLS)

The SLS experiment was carried out on a liquid chromatography apparatus (Wyatt Technology Corp.) consisting of an HPLC system (Agilent Technologies) connected in series with a triple-angle laser light scattering detector (miniDAWN TREOS), a UV detector at 280 nm (Agilent technologies), and a refractive index detector (RI-101, Shodex). 100 μl of purified BILBO1-NTD’ and FPC4-B1BD were mixed (molar ratio ~1:2, total 2 mg/ml) and, after 30-min incubation at room temperature, eluted from a Superdex 200 10/300 GL column (GE healthcare) at a flow rate of 0.5 ml per min. Data analysis was carried out using the Astra software (Wyatt technology).

### Isothermal titration calorimetry (ITC)

Purified BILBO1-NTD’ and FPC4-B1BD were dialysed overnight against a buffer containing 20 mM Tris-HCl (pH 8.0) and 50 mM NaCl. Protein concentration was determined by ND-1000 spectrophotometer (PEQlab). ITC experiments were carried out at 25°C using an iTC200 Microcalorimeter (MicroCal, GE healthcare). The cell contained 200 μl of 50 μM BILBO1-NTD’, which was titrated with an initial 0.4 μl injection followed by 19 constitutive injections (2 μl each) of 600 μM FPC4-B1BD with duration of 0.8 s. The interval between each two injections was 150 s. The ITC data were analysed using the program Origin version 7.0 provided by MicroCal. One-site binding model was used to fit the integrated data to calculate the stoichiometry and binding constants.

## Supporting information

S1 Fig Specificity of the rat anti-FPC4 antibody tested by western-blot. (A) Bacterial extracts (5x10^6^ bacteria/well) of non-induced (0h) or induced 2 hours (2h) for the expression of _6His_FPC4_6His_, and WC extracts (5x10^6^ *T*. *brucei* cells/well) of WT PCF or PCF non-induced (0h) and induced (48h) for the expression of myc-FPC4 were probed with the pre-immune serum (dilution 1:500). (B) The same samples as in A were probed with the immune serum. Anti-FPC4 detected a band corresponding to the expected size in induced bacteria (arrow) and induced PCF (*). (C) Anti-FPC4 labels specifically the purified histidine-tagged FPC4 protein aa 1–260 (30.41 kDa) but does not recognize the purified histidine-tagged FPC4 aa 357–440 (11.46 kDa). 20 ng of purified protein was loaded in each lane. (D) 5x10^6^ *T*. *brucei* WT cells expressing endogenous myc-tag FPC4 or cells over-expressing myc-FPC4 were probed with anti-myc. In D, using the anti-myc antibody, FPC4 is detectable by WB only when over-expressed. The monoclonal antibody mAb25, raised against *Tb*SAXO (an axoneme protein) was used as loading control. (E) The anti-TY1 antibody (BB2) is able to detect by western blot the 10xTY1 endogenously tagged FPC4 expressed in SmOxP427 (cell extract of 5.10^6^ cells) and over-expression is not required.
(TIF)

S2 Fig RNAi knockdown of FPC4 in PCF is not lethal. (A) Semi-quantitative RT-PCR on total RNA extracted from WT, FPC4^*RNAi*^ non-induced (0), and FPC4^*RNAi*^ induced cells at 1 to 10 days of induction. (B) Growth curve of WT, FPC4^*RNAi*^ non-induced and FPC4^*RNAi*^ induced cells. Error bars represent the standard error from 3 independent experiments (and are smaller than the data point mark). (C) Detection of FPC4 using anti-FPC4 on cytoskeleton extracted FPC4^*RNAi*^ cells non-induced or induced for 48h. Scale bar represents 5 μm.
(TIF)

S3 Fig Amino acid sequence and alignment of FPC4 1–217 and shuffled FPC4 1–217 using Clustal Omega [81]. (PDF)

S4 Fig MORN1 is present in the FPC connecting fibre of FPC4 ΔB1BD over-expressing cell line. Immuno-gold electron microscopy localisation of myc-FPC4-ΔB1BD (anti-myc, 15 nm gold) and MORN11 (anti-MORN1, 10 nm gold) on isolated flagella from cells expressing myc-FPC4-ΔB1BD.
(TIF)

## References

[1] Bornens M. Organelle positioning and cell polarity. Nat Rev Mol Cell Biol. 2008;9: 874–86. doi: 10.1038/nrm2524 18946476 10.1038/nrm2524

[2] Rafelski SM, Marshall WF. Building the cell: design principles of cellular architecture. Nat Rev Mol Cell Biol. 2008;9: 593–602. doi: 10.1038/nrm2460 18648373 10.1038/nrm2460

[3] Steverding D. The history of African trypanosomiasis. Parasit Vectors. 2008;1: 3 doi: 10.1186/1756-3305-1-3 18275594 10.1186/1756-3305-1-3 PMC2270819

[4] Robinson DR, Sherwin T, Ploubidou A, Byard EH, Gull K. Microtubule polarity and dynamics in the control of organelle positioning, segregation, and cytokinesis in the trypanosome cell cycle. J Cell Biol. 1995;128: 1163–72. 7896879 10.1083/jcb.128.6.1163 PMC2120423

[5] Hammarton TC, Kramer S, Tetley L, Boshart M, Mottram JC. Trypanosoma brucei Polo-like kinase is essential for basal body duplication, kDNA segregation and cytokinesis. Mol Microbiol. 2007;65: 1229–48. doi: 10.1111/j.1365-2958.2007.05866.x 17662039 10.1111/j.1365-2958.2007.05866.x PMC2169650

[6] Lacomble S, Vaughan S, Gadelha C, Morphew MK, Shaw MK, McIntosh JR, et al Basal body movements orchestrate membrane organelle division and cell morphogenesis in Trypanosoma brucei. J Cell Sci. 2010;123: 2884–2891. doi: 10.1242/jcs.074161 20682637 10.1242/jcs.074161 PMC2923567

[7] Hughes L, Borrett S, Towers K, Starborg T, Vaughan S. Patterns of organelle ontogeny through a cell cycle revealed by whole cell reconstructions using 3D electron microscopy. J Cell Sci. 2017; jcs.198887. doi: 10.1242/jcs.198887 28049718 10.1242/jcs.198887

[8] Vickerman K. The mechanism of cyclical development in trypanosomes of the Trypanosoma brucei sub-group: an hypothesis based on ultrastructural observations. Trans R Soc Trop Med Hyg. 1962;56: 487–495. 13997060 10.1016/0035-9203(62)90072-x

[9] Hoare CA, Wallace FG. Developmental Stages of Trypanosomatid Flagellates: a New Terminology. Nature. 1966;212: 1385–1386. doi: 10.1038/2121385a0

[10] Matthews KR. Controlling and Coordinating Development in Vector-Transmitted Parasites. Science. 2011;331: 1149–1153. doi: 10.1126/science.1198077 21385707 10.1126/science.1198077 PMC3834321

[11] McKean PG. Coordination of cell cycle and cytokinesis in Trypanosoma brucei. Curr Opin Microbiol. 2003;6: 600–7. 14662356 10.1016/j.mib.2003.10.010

[12] Gull K. The cytoskeleton of trypanosomatid parasites. Annu Rev Microbiol. 1999;53: 629–55. doi: 10.1146/annurev.micro.53.1.629 10547703 10.1146/annurev.micro.53.1.629

[13] Robinson DR, Gull K. Basal body movements as a mechanism for mitochondrial genome segregation in the trypanosome cell cycle. Nature. 1991;352: 731–3. doi: 10.1038/352731a0 1876188 10.1038/352731a0

[14] Ogbadoyi EO, Robinson DR, Gull K. A high-order trans-membrane structural linkage is responsible for mitochondrial genome positioning and segregation by flagellar basal bodies in trypanosomes. Mol Biol Cell. 2003;14: 1769–79. doi: 10.1091/mbc.E02-08-0525 12802053 10.1091/mbc.E02-08-0525 PMC165075

[15] Sunter JD, Gull K. The Flagellum Attachment Zone: ‘The Cellular Ruler’ of Trypanosome Morphology. Trends Parasitol. 2016;32: 309–324. doi: 10.1016/j.pt.2015.12.010 26776656 10.1016/j.pt.2015.12.010 PMC4827413

[16] Vaughan S, Kohl L, Ngai I, Wheeler RJ, Gull K. A repetitive protein essential for the flagellum attachment zone filament structure and function in Trypanosoma brucei. Protist. 2008;159: 127–36. doi: 10.1016/j.protis.2007.08.005 17945531 10.1016/j.protis.2007.08.005

[17] Taylor AER, Godfrey DG. A New Organelle of Bloodstream Salivarian Trypanosomes. J Protozool. 1969;16: 466–470. doi: 10.1111/j.1550-7408.1969.tb02302.x 5343461 10.1111/j.1550-7408.1969.tb02302.x

[18] Vickerman K. On The Surface Coat and Flagellar Adhesion in Trypanosomes. J Cell Sci. 1969;5: 163–193. 5353653 10.1242/jcs.5.1.163

[19] Lacomble S, Vaughan S, Gadelha C, Morphew MK, Shaw MK, McIntosh JR, et al Three-dimensional cellular architecture of the flagellar pocket and associated cytoskeleton in trypanosomes revealed by electron microscope tomography. J Cell Sci. 2009;122: 1081–90. doi: 10.1242/jcs.045740 19299460 10.1242/jcs.045740 PMC2714436

[20] Allen CL, Goulding D, Field MC. Clathrin-mediated endocytosis is essential in Trypanosoma brucei. EMBO J. 2003;22: 4991–5002. doi: 10.1093/emboj/cdg481 14517238 10.1093/emboj/cdg481 PMC204465

[21] Engstler M, Thilo L, Weise F, Grunfelder CG, Schwarz H, Boshart M, et al Kinetics of endocytosis and recycling of the GPI-anchored variant surface glycoprotein in Trypanosoma brucei. J Cell Sci. 2004;117: 1105–15. doi: 10.1242/jcs.00938 14996937 10.1242/jcs.00938

[22] Hall BS, Gabernet-Castello C, Voak A, Goulding D, Natesan SK, Field MC. TbVps34, the trypanosome orthologue of Vps34, is required for Golgi complex segregation. J Biol Chem. 2006;281: 27600–27612. doi: 10.1074/jbc.M602183200 16835237 10.1074/jbc.M602183200

[23] Engstler M, Pfohl T, Herminghaus S, Boshart M, Wiegertjes G, Heddergott N, et al Hydrodynamic flow-mediated protein sorting on the cell surface of trypanosomes. Cell. 2007;131: 505–15. doi: 10.1016/j.cell.2007.08.046 17981118 10.1016/j.cell.2007.08.046

[24] Sherwin T, Gull K. The cell division cycle of Trypanosoma brucei brucei: timing of event markers and cytoskeletal modulations. Philos Trans R Soc Lond B Biol Sci. 1989;323: 573–88. 2568647 10.1098/rstb.1989.0037

[25] Bonhivers M, Nowacki S, Landrein N, Robinson DR. Biogenesis of the trypanosome endo-exocytotic organelle is cytoskeleton mediated. PLoS Biol. 2008;6: e105 doi: 10.1371/journal.pbio.0060105 18462016 10.1371/journal.pbio.0060105 PMC2365980

[26] He CY, Ho HH, Malsam J, Chalouni C, West CM, Ullu E, et al Golgi duplication in Trypanosoma brucei. J Cell Biol. 2004;165: 313–21. doi: 10.1083/jcb.200311076 15138289 10.1083/jcb.200311076 PMC2172185

[27] Yelinek JT, He CY, Warren G. Ultrastructural Study of Golgi Duplication in Trypanosoma brucei. Traffic. 2009;10: 300–306. doi: 10.1111/j.1600-0854.2008.00873.x 19207482 10.1111/j.1600-0854.2008.00873.x

[28] He CY, Pypaert M, Warren G. Golgi Duplication in Trypanosoma brucei Requires Centrin2. Science. 2005;310: 1196–1198. doi: 10.1126/science.1119969 16254149 10.1126/science.1119969

[29] Morriswood B. Form, Fabric, and Function of a Flagellum-Associated Cytoskeletal Structure. Cells. 2015;4: 726–747. doi: 10.3390/cells4040726 26540076 10.3390/cells4040726 PMC4695855

[30] Selvapandiyan A, Kumar P, Morris JC, Salisbury JL, Wang CC, Nakhasi HL. Centrin1 Is Required for Organelle Segregation and Cytokinesis in Trypanosoma brucei. Mol Biol Cell. 2007;18: 3290–3301. doi: 10.1091/mbc.E07-01-0022 17567955 10.1091/mbc.E07-01-0022 PMC1951761

[31] Shi J, Franklin JB, Yelinek JT, Ebersberger I, Warren G, He CY. Centrin4 coordinates cell and nuclear division in T. brucei. J Cell Sci. 2008;121: 3062–70. doi: 10.1242/jcs.030643 18768932 10.1242/jcs.030643

[32] de Graffenried CL, Ho HH, Warren G. Polo-like kinase is required for Golgi and bilobe biogenesis in *Trypanosoma brucei*. J Cell Biol. 2008;181: 431–438. doi: 10.1083/jcb.200708082 18443217 10.1083/jcb.200708082 PMC2364693

[33] André J, Harrison S, Towers K, Qi X, Vaughan S, McKean PG, et al The tubulin cofactor C family member TBCCD1 orchestrates cytoskeletal filament formation. J Cell Sci. 2013;126: 5350–5356. doi: 10.1242/jcs.136515 24101722 10.1242/jcs.136515

[34] Morriswood B, Havlicek K, Demmel L, Yavuz S, Sealey-Cardona M, Vidilaseris K, et al Novel Bilobe Components in Trypanosoma brucei Identified Using Proximity-Dependent Biotinylation. Eukaryot Cell. 2013;12: 356–367. doi: 10.1128/EC.00326-12 23264645 10.1128/EC.00326-12 PMC3571296

[35] Dean S, Moreira-Leite F, Varga V, Gull K. Cilium transition zone proteome reveals compartmentalization and differential dynamics of ciliopathy complexes. Proc Natl Acad Sci. 2016;113: E5135–E5143. doi: 10.1073/pnas.1604258113 27519801 10.1073/pnas.1604258113 PMC5024643

[36] McAllaster MR, Ikeda KN, Lozano-Núñez A, Anrather D, Unterwurzacher V, Gossenreiter T, et al Proteomic identification of novel cytoskeletal proteins associated with TbPLK, an essential regulator of cell morphogenesis in Trypanosoma brucei. Mol Biol Cell. 2015;26: 3013–3029. doi: 10.1091/mbc.E15-04-0219 26133384 10.1091/mbc.E15-04-0219 PMC4551316

[37] Hu H, Zhou Q, Li Z. SAS-4 Protein in Trypanosoma brucei Controls Life Cycle Transitions by Modulating the Length of the Flagellum Attachment Zone Filament. J Biol Chem. 2015;290: 30453–30463. doi: 10.1074/jbc.M115.694109 26504079 10.1074/jbc.M115.694109 PMC4683267

[38] Dang HQ, Zhou Q, Rowlett VW, Hu H, Lee KJ, Margolin W, et al Proximity Interactions among Basal Body Components in Trypanosoma brucei Identify Novel Regulators of Basal Body Biogenesis and Inheritance. mBio. 2017;8: e02120–16. doi: 10.1128/mBio.02120-16 28049148 10.1128/mBio.02120-16 PMC5210500

[39] Zhou Q, Gheiratmand L, Chen Y, Lim TK, Zhang J, Li S, et al A comparative proteomic analysis reveals a new bi-lobe protein required for bi-lobe duplication and cell division in Trypanosoma brucei. PLoS One. 2010;5: e9660 doi: 10.1371/journal.pone.0009660 20300570 10.1371/journal.pone.0009660 PMC2837748

[40] Brasseur A, Bayat S, Chua XL, Zhang Y, Zhou Q, Low BC, et al The bi-lobe-associated LRRP1 regulates Ran activity in Trypanosoma brucei. J Cell Sci. 2014;127: 4846–4856. doi: 10.1242/jcs.148015 25217630 10.1242/jcs.148015

[41] Morriswood B, He CY, Sealey-Cardona M, Yelinek J, Pypaert M, Warren G. The bilobe structure of Trypanosoma brucei contains a MORN-repeat protein. Mol Biochem Parasitol. 2009;167: 95–103. doi: 10.1016/j.molbiopara.2009.05.001 19445968 10.1016/j.molbiopara.2009.05.001

[42] Morriswood B, Schmidt K. A MORN Repeat Protein Facilitates Protein Entry into the Flagellar Pocket of Trypanosoma brucei. Eukaryot Cell. 2015;14: 1081–1093. doi: 10.1128/EC.00094-15 26318396 10.1128/EC.00094-15 PMC4621311

[43] Esson HJ, Morriswood B, Yavuz S, Vidilaseris K, Dong G, Warren G. Morphology of the Trypanosome Bilobe, a Novel Cytoskeletal Structure. Eukaryot Cell. 2012;11: 761–772. doi: 10.1128/EC.05287-11 22327007 10.1128/EC.05287-11 PMC3370457

[44] Florimond C, Sahin A, Vidilaseris K, Dong G, Landrein N, Dacheux D, et al BILBO1 Is a Scaffold Protein of the Flagellar Pocket Collar in the Pathogen Trypanosoma brucei. PLoS Pathog. 2015;11: e1004654 doi: 10.1371/journal.ppat.1004654 25822645 10.1371/journal.ppat.1004654 PMC4379179

[45] Vidilaseris K, Morriswood B, Kontaxis G, Dong G. Structure of the TbBILBO1 protein N-terminal domain from Trypanosoma brucei reveals an essential requirement for a conserved surface patch. J Biol Chem. 2014;289: 3724–35. doi: 10.1074/jbc.M113.529032 24362019 10.1074/jbc.M113.529032 PMC3916570

[46] Vidilaseris K, Shimanovskaya E, Esson HJ, Morriswood B, Dong G. Assembly mechanism of Trypanosoma brucei BILBO1, a multidomain cytoskeletal protein. J Biol Chem. 2014;289: 23870–23881. doi: 10.1074/jbc.M114.554659 25031322 10.1074/jbc.M114.554659 PMC4156054

[47] Lupas A, Van Dyke M, Stock J. Predicting coiled coils from protein sequences. Science. 1991;252: 1162–4. doi: 10.1126/science.252.5009.1162 2031185 10.1126/science.252.5009.1162

[48] Kosugi S, Hasebe M, Tomita M, Yanagawa H. Systematic identification of cell cycle-dependent yeast nucleocytoplasmic shuttling proteins by prediction of composite motifs. Proc Natl Acad Sci. 2009;106: 10171–10176. doi: 10.1073/pnas.0900604106 19520826 10.1073/pnas.0900604106 PMC2695404

[49] Amos LA, Schlieper D. Microtubules and maps. Adv Protein Chem. 2005;71: 257–98. doi: 10.1016/S0065-3233(04)71007-4 16230114 10.1016/S0065-3233(04)71007-4

[50] Perdomo D, Bonhivers M, Robinson DR. The Trypanosome Flagellar Pocket Collar and Its Ring Forming Protein—TbBILBO1. Cells. 2016;5: 9 doi: 10.3390/cells5010009 26950156 10.3390/cells5010009 PMC4810094

[51] Kumar P, Wang CC. Dissociation of Cytokinesis Initiation from Mitotic Control in a Eukaryote. Eukaryot Cell. 2006;5: 92–102. doi: 10.1128/EC.5.1.92-102.2006 16400171 10.1128/EC.5.1.92-102.2006 PMC1360254

[52] Hu H, Zhou Q, Han X, Li Z. CRL4WDR1 Controls Polo-like Kinase Protein Abundance to Promote Bilobe Duplication, Basal Body Segregation and Flagellum Attachment in Trypanosoma brucei. PLOS Pathog. 2017;13: e1006146 doi: 10.1371/journal.ppat.1006146 28052114 10.1371/journal.ppat.1006146 PMC5241021

[53] Alsford S, Turner DJ, Obado SO, Sanchez-Flores A, Glover L, Berriman M, et al High-throughput phenotyping using parallel sequencing of RNA interference targets in the African trypanosome. Genome Res. 2011;21: 915–24. doi: 10.1101/gr.115089.110 21363968 10.1101/gr.115089.110 PMC3106324

[54] Ikeda KN, Graffenried CL de. Polo-like kinase is necessary for flagellum inheritance in Trypanosoma brucei. J Cell Sci. 2012;125: 3173–3184. doi: 10.1242/jcs.101162 22427687 10.1242/jcs.101162

[55] Gheiratmand L, Brasseur A, Zhou Q, He CY. Biochemical characterization of the bi-lobe reveals a continuous structural network linking the bi-lobe to other single-copied organelles in Trypanosoma brucei. J Biol Chem. 2013;288: 3489–99. doi: 10.1074/jbc.M112.417428 23235159 10.1074/jbc.M112.417428 PMC3561568

[56] Hayes P, Varga V, Olego-Fernandez S, Sunter J, Ginger ML, Gull K. Modulation of a cytoskeletal calpain-like protein induces major transitions in trypanosome morphology. J Cell Biol. 2014;206: 377–84. doi: 10.1083/jcb.201312067 25092656 10.1083/jcb.201312067 PMC4121973

[57] Sasse R, Gull K. Tubulin post-translational modifications and the construction of microtubular organelles in Trypanosoma brucei. J Cell Sci. 1988;90: 577–589. 3075618 10.1242/jcs.90.4.577

[58] de Graffenried CL, Anrather D, Von Raussendorf F, Warren G. Polo-like kinase phosphorylation of bilobe-resident TbCentrin2 facilitates flagellar inheritance in Trypanosoma brucei. Mol Biol Cell. 2013;24: 1947–63. doi: 10.1091/mbc.E12-12-0911 23615446 10.1091/mbc.E12-12-0911 PMC3681699

[59] Hirano Y, Yoshinaga S, Takeya R, Suzuki NN, Horiuchi M, Kohjima M, et al Structure of a Cell Polarity Regulator, a Complex between Atypical PKC and Par6 PB1 Domains. J Biol Chem. 2005;280: 9653–9661. doi: 10.1074/jbc.M409823200 15590654 10.1074/jbc.M409823200

[60] Gopalakrishnan S, Hallett MA, Atkinson SJ, Marrs JA. aPKC-PAR complex dysfunction and tight junction disassembly in renal epithelial cells during ATP depletion. Am J Physiol Cell Physiol. 2007;292: C1094–1102. doi: 10.1152/ajpcell.00099.2006 16928777 10.1152/ajpcell.00099.2006

[61] Chen S, Chen J, Shi H, Wei M, Castaneda-Castellanos DR, Bultje RS, et al Regulation of microtubule stability and organization by mammalian Par3 in specifying neuronal polarity. Dev Cell. 2013;24: 26–40. doi: 10.1016/j.devcel.2012.11.014 23273878 10.1016/j.devcel.2012.11.014 PMC3549028

[62] Chen J, Zhang M. The Par3/Par6/aPKC complex and epithelial cell polarity. Exp Cell Res. 2013;319: 1357–1364. doi: 10.1016/j.yexcr.2013.03.021 23535009 10.1016/j.yexcr.2013.03.021

[63] Graham TM, Tait A, Hide G. Characterisation of a polo-like protein kinase gene homologue from an evolutionary divergent eukaryote, Trypanosoma brucei. Gene. 1998;207: 71–77. doi: 10.1016/S0378-1119(97)00606-9 9511745 10.1016/s0378-1119(97)00606-9

[64] Werner ME, Mitchell JW, Putzbach W, Bacon E, Kim SK, Mitchell BJ. Radial intercalation is regulated by the Par complex and the microtubule-stabilizing protein CLAMP/Spef1. J Cell Biol. 2014;206: 367–376. doi: 10.1083/jcb.201312045 25070955 10.1083/jcb.201312045 PMC4121976

[65] Durante IM, Cámara M de los M, Buscaglia CA. A Novel Trypanosoma cruzi Protein Associated to the Flagellar Pocket of Replicative Stages and Involved in Parasite Growth. PLOS ONE. 2015;10: e0130099 doi: 10.1371/journal.pone.0130099 26086767 10.1371/journal.pone.0130099 PMC4472858

[66] Wheeler RJ, Sunter JD, Gull K. Flagellar pocket restructuring through the Leishmania life cycle involves a discrete flagellum attachment zone. J Cell Sci. 2016;129: 854–867. doi: 10.1242/jcs.183152 26746239 10.1242/jcs.183152 PMC4760377

[67] Melville SE, Leech V, Navarro M, Cross GAM. The molecular karyotype of the megabase chromosomes of Trypanosoma brucei stock 427. Mol Biochem Parasitol. 2000;111: 261–273. doi: 10.1016/S0166-6851(00)00316-9 11163435 10.1016/s0166-6851(00)00316-9

[68] Wirtz E, Clayton C. Inducible gene expression in trypanosomes mediated by a prokaryotic repressor. Science. 1995;268: 1179–1183. doi: 10.1126/science.7761835 7761835 10.1126/science.7761835

[69] Wirtz E, Leal S, Ochatt C, Cross GA. A tightly regulated inducible expression system for conditional gene knock-outs and dominant-negative genetics in Trypanosoma brucei. Mol Biochem Parasitol. 1999;99: 89–101. 10215027 10.1016/s0166-6851(99)00002-x

[70] Schumann Burkard G, Jutzi P, Roditi I. Genome-wide RNAi screens in bloodstream form trypanosomes identify drug transporters. Mol Biochem Parasitol. 2011;175: 91–94. doi: 10.1016/j.molbiopara.2010.09.002 20851719 10.1016/j.molbiopara.2010.09.002

[71] Dean S, Sunter J, Wheeler RJ, Hodkinson I, Gluenz E, Gull K. A toolkit enabling efficient, scalable and reproducible gene tagging in trypanosomatids. Open Biol. 2015;5: 140197 doi: 10.1098/rsob.140197 25567099 10.1098/rsob.140197 PMC4313374

[72] Hirumi H, Hirumi K. Continuous cultivation of Trypanosoma brucei blood stream forms in a medium containing a low concentration of serum protein without feeder cell layers. J Parasitol. 1989;75: 985–9. 2614608

[73] Heldin CH, Johnsson A, Wennergren S, Wernstedt C, Betsholtz C, Westermark B. A human osteosarcoma cell line secretes a growth factor structurally related to a homodimer of PDGF A-chains. Nature. 1986;319: 511–4. doi: 10.1038/319511a0 3456080 10.1038/319511a0

[74] Clayton CE, Estevez AM, Hartmann C, Alibu VP, Field M, Horn D. Down-regulating gene expression by RNA interference in Trypanosoma brucei. Methods Mol Biol. 2005;309: 39–60. doi: 10.1385/1-59259-935-4:039 15990397 10.1385/1-59259-935-4:039

[75] Oberholzer M, Morand S, Kunz S, Seebeck T. A vector series for rapid PCR-mediated C-terminal in situ tagging of Trypanosoma brucei genes. Mol Biochem Parasitol. 2006;145: 117–20. doi: 10.1016/j.molbiopara.2005.09.002 16269191 10.1016/j.molbiopara.2005.09.002

[76] Brenndörfer M, Boshart M. Selection of reference genes for mRNA quantification in Trypanosoma brucei. Mol Biochem Parasitol. 2010;172: 52–55. doi: 10.1016/j.molbiopara.2010.03.007 20302889 10.1016/j.molbiopara.2010.03.007

[77] Kohl L, Sherwin T, Gull K. Assembly of the paraflagellar rod and the flagellum attachment zone complex during the Trypanosoma brucei cell cycle. J Eukaryot Microbiol. 1999;46: 105–9. 10361731 10.1111/j.1550-7408.1999.tb04592.x

[78] Bastin P, Bagherzadeh Z, Matthews KR, Gull K. A novel epitope tag system to study protein targeting and organelle biogenesis in Trypanosoma brucei. Mol Biochem Parasitol. 1996;77: 235–9. 8813669 10.1016/0166-6851(96)02598-4

[79] Dacheux D, Roger B, Bosc C, Landrein N, Roche E, Chansel L, et al Human SAXO1 (FAM154A) is a microtubule-stabilizing protein specific to cilia and related structures. J Cell Sci. 2015; jcs.155143. doi: 10.1242/jcs.155143 25673876 10.1242/jcs.155143

[80] Dacheux D, Landrein N, Thonnus M, Gilbert G, Sahin A, Wodrich H, et al A MAP6-Related Protein Is Present in Protozoa and Is Involved in Flagellum Motility. PLoS One. 2012;7: e31344 doi: 10.1371/journal.pone.0031344 22355359 10.1371/journal.pone.0031344 PMC3280300

[81] Sievers F, Wilm A, Dineen D, Gibson TJ, Karplus K, Li W, et al Fast, scalable generation of high-quality protein multiple sequence alignments using Clustal Omega. Mol Syst Biol. 2011;7: 539 doi: 10.1038/msb.2011.75 21988835 10.1038/msb.2011.75 PMC3261699

