# Supplementary figures and images for: Interaction between the flagellar pocket collar and the hook complex via a novel microtubule-binding protein in Trypanosoma brucei
Source: PLoS Pathog. 2017 Nov 1;13(11):e1006710. doi: 10.1371/journal.ppat.1006710 (PMC5683654; doi:10.1371/journal.ppat.1006710)

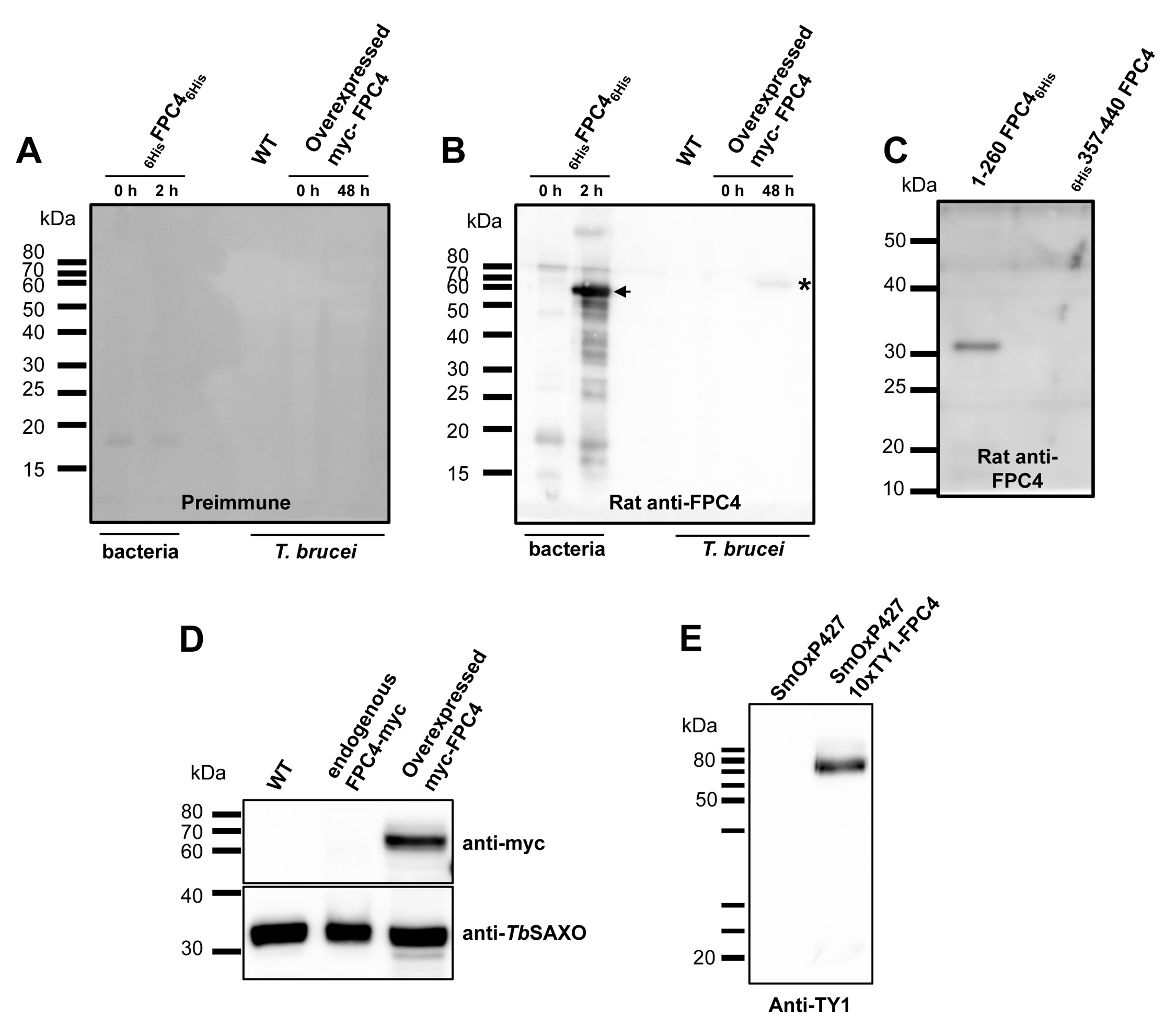

Supplement: S1 Fig — (A) Bacterial extracts (5x10^6^ bacteria/well) of non-induced (0h) or induced 2 hours (2h) for the expression of 6HisFPC46His, and WC extracts (5x10^6^ T. brucei cells/well) of WT PCF or PCF non-induced (0h) and induced (48h) for the expression of myc-FPC4 were probed with the pre-immune serum (dilution 1:500). (B) The same samples as in A were probed with the immune serum. Anti-FPC4 detected a band corresponding to the expected size in induced bacteria (arrow) and induced PCF (*). (C) Anti-FPC4 labels specifically the purified histidine-tagged FPC4 protein aa 1–260 (30.41 kDa) but does not recognize the purified histidine-tagged FPC4 aa 357–440 (11.46 kDa). 20 ng of purified protein was loaded in each lane. (D) 5x10^6^ T. brucei WT cells expressing endogenous myc-tag FPC4 or cells over-expressing myc-FPC4 were probed with anti-myc. In D, using the anti-myc antibody, FPC4 is detectable by WB only when over-expressed. The monoclonal antibody mAb25, raised against TbSAXO (an axoneme protein) was used as loading control. (E) The anti-TY1 antibody (BB2) is able to detect by western blot the 10xTY1 endogenously tagged FPC4 expressed in SmOxP427 (cell extract of 5.106 cells) and over-expression is not required. (TIF) [file ppat.1006710.s001.tif]

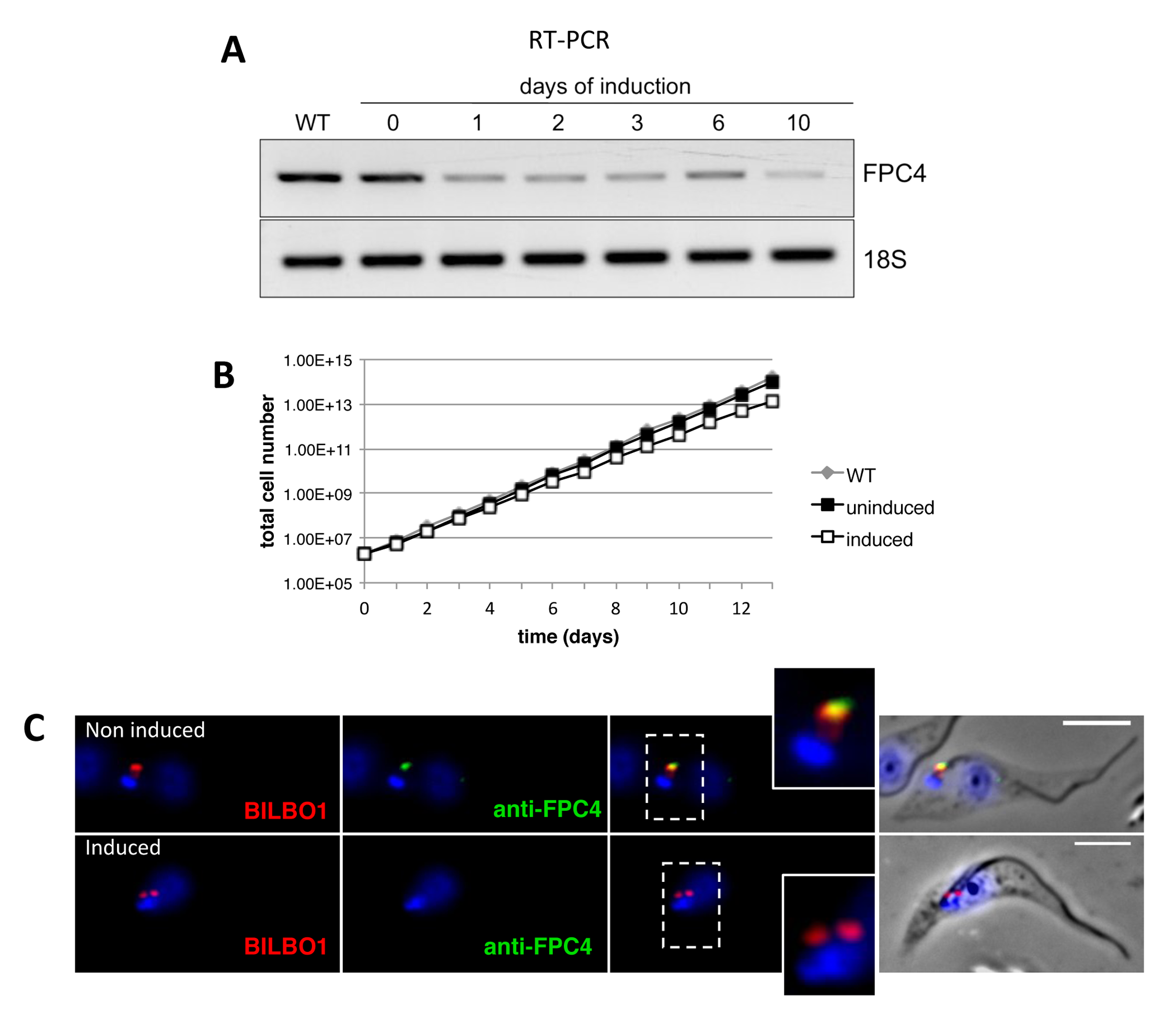

Supplement: S2 Fig — (A) Semi-quantitative RT-PCR on total RNA extracted from WT, FPC4RNAi non-induced (0), and FPC4RNAi induced cells at 1 to 10 days of induction. (B) Growth curve of WT, FPC4RNAi non-induced and FPC4RNAi induced cells. Error bars represent the standard error from 3 independent experiments (and are smaller than the data point mark). (C) Detection of FPC4 using anti-FPC4 on cytoskeleton extracted FPC4RNAi cells non-induced or induced for 48h. Scale bar represents 5 μm. (TIF) [file ppat.1006710.s002.tif]

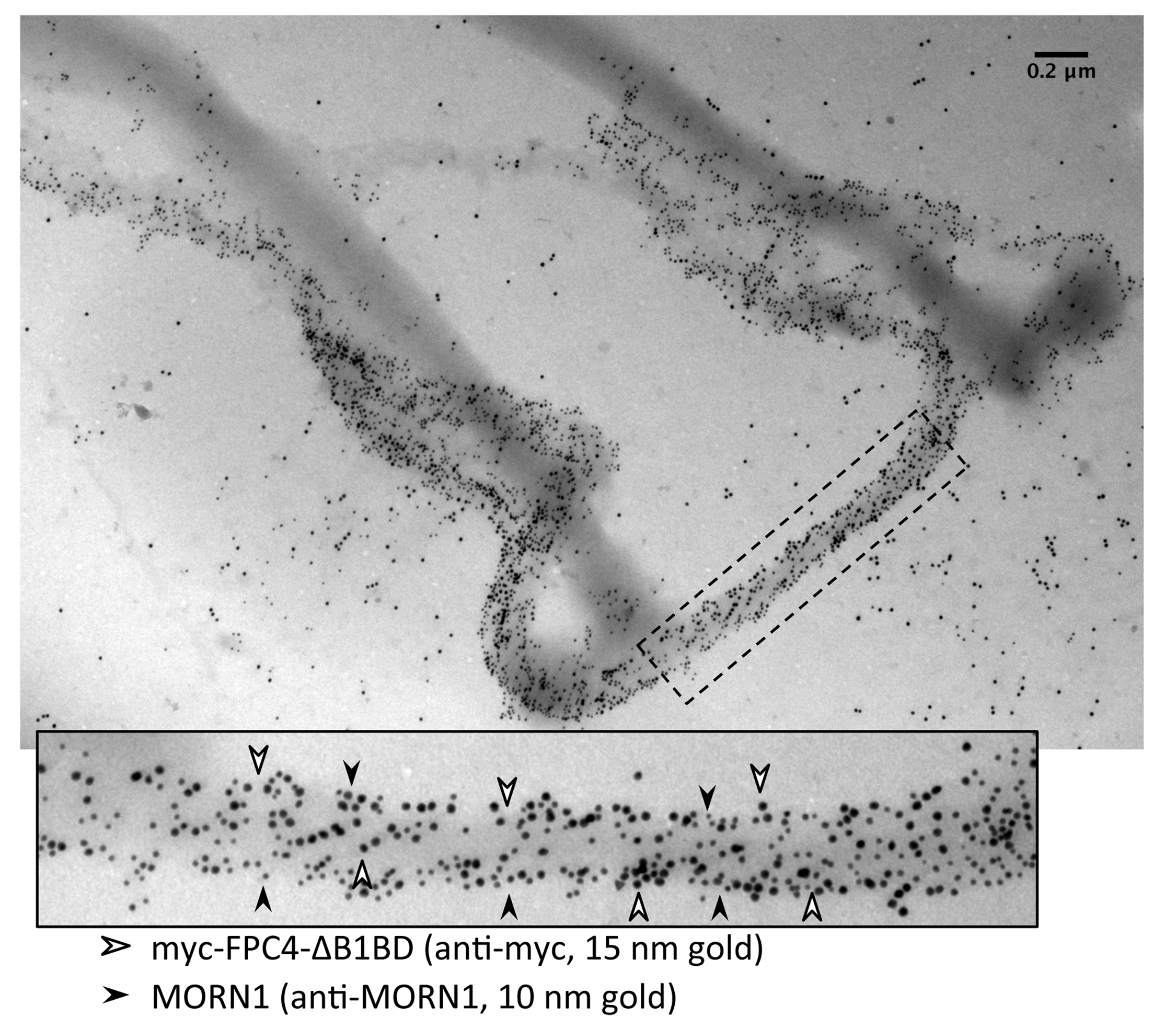

Supplement: S4 Fig — Immuno-gold electron microscopy localisation of myc-FPC4-ΔB1BD (anti-myc, 15 nm gold) and MORN11 (anti-MORN1, 10 nm gold) on isolated flagella from cells expressing myc-FPC4-ΔB1BD. (TIF) [file ppat.1006710.s004.tif]
