# Supplementary material for: Interaction between the flagellar pocket collar and the hook complex via a novel microtubule-binding protein in Trypanosoma brucei
Source: PLoS Pathog. 2017 Nov 1;13(11):e1006710. doi: 10.1371/journal.ppat.1006710 (PMC5683654; doi:10.1371/journal.ppat.1006710)
Supplement: S3 Fig — (PDF) [file ppat.1006710.s003.pdf]

Albisetti et al, S3 Fig.

```
>FPC4_aa 1-217
MKEKKEVNSSRKGWMPPRGRLTATSRGPVPPPPSRTPTRCSSICRVPKDRFRGPPAVAATTTPRSRESGSACHCVPLRR
KNASNDNAFATRRTAIVDEKTPSAQKPVQPYVADERIEKMRRSMNGTNRRSCSESMRDAEPVSFHMAPYPRVPFVRQSS
NRCESTPSSYRTRVSTAQQCSFGGHAARPTVAGTELLPRMPHLQGTHRTSKERGRATLE
>Shuffled_aa 1-217
MHPSFSEERSNNHKYRGNRKAVARRHNPDARTKECPKTTSTTSRTSPTTLSTPSYEPAPDCIPQGGLDLGSSERERRAA
PSKSRSNNGMGPSRKLRARRAKRRDSTVTPVPTASPVGHMGPPTMVVFASSVADRSTRRACWCSASQVLPHPRRRQKSV
TAAPAESEIRCPRSRPQMQVNTVETVRPFARIAGFEPRCVQRKEPRTMEGKGPCYLFMT


Aligned_sequences: 2
# 1: Tb927.8.6370_1-217
# 2: Shuffled_1-217
# Matrix: EBLOSUM62
# Gap_penalty: 10.0
# Extend_penalty: 0.5
# Length: 311
# Identity:      33/311 (10.6%)
# Similarity:    49/311 (15.8%)
# Gaps:         188/311 (60.5%)
# Score: 39.5

Tb927.8.6370       1 --------------------------------------------------      0
Shuffled_1-217     1 MHPSFSEERSNNHKYRGNRKAVARRHNPDARTKECPKTTSTTSRTSPTTL     50

Tb927.8.6370       1 ----------------------MKEKKEVNS---SRKGWMPP---RGRLT     22
                                           .:|::...|   |..|..|.   |.|..
Shuffled_1-217    51 STPSYEPAPDCIPQGGLDLGSSERERRAAPSKSRSNNGMGPSRKLRARRA    100

Tb927.8.6370      23 ATSRGPVPPPPSRTPTRCSSICRVPKDRFRGPPA-VAATTTPRSRESGSA     71
                     ......|.|.|:.:|.           ...|||. |...::...|.:..|
Shuffled_1-217   101 KRRDSTVTPVPTASPV-----------GHMGPPTMVVFASSVADRSTRRA    139

Tb927.8.6370      72 CHC-----VPLRRKNASNDNAFATRRTAIVDEKTPSAQ----KPVQPYVA    112
                     |.|     :|..|:...:..|.............|..|    :.|:|:..
Shuffled_1-217   140 CWCSASQVLPHPRRRQKSVTAAPAESEIRCPRSRPQMQVNTVETVRPFAR    189

Tb927.8.6370     113 DERIE------KMRRSMNGTNRRSCSESMRDAEPVSFHMAPYPRVPFVRQ    156
                     ....|      |..|:|.|  :..|...|.
Shuffled_1-217   190 IAGFEPRCVQRKEPRTMEG--KGPCYLFMT--------------------    217

Tb927.8.6370     157 SSNRCESTPSSYRTRVSTAQQCSFGGHAARPTVAGTELLPRMPHLQGTHR    206
Shuffled_1-217   218 --------------------------------------------------    217

Tb927.8.6370     207 TSKERGRATLE    217
Shuffled_1-217   218 -----------    217
```
